# Deep intraspecific DNA barcode splits and hybridisation in the *Udea alpinalis* group (Insecta, Lepidoptera, Crambidae) – an integrative revision

**DOI:** 10.3897/zookeys.746.22020

**Published:** 2018-03-13

**Authors:** Richard Mally, Peter Huemer, Matthias Nuss

**Affiliations:** 1 University Museum of Bergen, Natural History Collections, Realfagbygget, Allégaten 41, 5007 Bergen, Norway; 2 Tiroler Landesmuseen Betriebsges.m.b.H., Natural History Department, Collections and Research Center, Krajnc-Str. 1, 6060 Hall, Austria; 3 Senckenberg Museum of Zoology, Königsbrücker Landstraße 159, 01109 Dresden, Germany

**Keywords:** Alps, Central Asia, montane, nuclear genes, introgression, *Wolbachia*

## Abstract

The analysis of mitochondrial COI data for the European-Centroasian montane *Udea alpinalis* species group finds deep intraspecific splits. Specimens of *U. austriacalis* and *U. rhododendronalis* separate into several biogeographical groups. These allopatric groups are not recovered in the analyses of the two nuclear markers wingless and Elongation factor 1-alpha, except for *U. austriacalis* from the Pyrenees and the French Massif Central. The latter populations are also morphologically distinct and conspecific with *Scopula donzelalis* Guenée, 1854, which is removed from synonymy and reinstated as *Udea donzelalis* (Guenée, 1854) **stat. rev.** Furthermore, *Udea altaica* (Zerny, 1914), **stat. n.** from the Mongolian central Altai mountains, *U. juldusalis* (Zerny, 1914), **stat. n.** from the Tian Shan mountains of Kazakhstan, Kyrgyzstan and NW China, and *U. plumbalis* (Zerny, 1914), **stat. n.** from the Sayan Mountains of Northern Mongolia are raised to species level, and lectotypes are designated. Evidence of introgression of *U. alpinalis* into *U. uliginosalis* at three localities in the Central Alps is presented. A screening for *Wolbachia* using the markers wsp, gatB and ftsZ was negative for the *U. alpinalis* species group, but *Wolbachia* was found in single specimens of *U. fulvalis* and *U. olivalis* (both in the *U. numeralis* species group). We do not find evidence for the conjecture of several authors of additional subspecies in *U. rhododendronalis*, and synonymise *U. rhododendronalis luquetalis* Leraut, 1996, **syn. n.** and *U. r. ventosalis* Leraut, 1996, **syn. n.** with the nominal *U. rhododendronalis* (Duponchel, 1834).

## Introduction

With currently 217 recognised species (Nuss et al. 2003–2018), *Udea* Guenée (in Duponchel), 1845 is the most diverse genus of Spilomelinae within Crambidae. Munroe (1966) revised the North American species of *Udea*. Mally and Nuss (2011) proposed a phylogenetic framework for the majority of the 39 currently recognised European species and described four monophyletic species groups: the *U. ferrugalis*, *U. itysalis*, *U. numeralis* and *U. alpinalis* species groups. Whereas the first three species groups are also represented on other continents, the *U. alpinalis* group occurs, to the present knowledge, only in the mountain systems from Europe to Central Asia. Currently, this group contains nine species: *Udea alpinalis* (Denis & Schiffermüller, 1775), *U. austriacalis* (Herrich-Schäffer, 1851), *U. bourgognealis* Leraut, 1996, *U. carniolica* Huemer & Tarmann, 1989, *U. cretacea* (Filipjev, 1925), *U. murinalis* (Fischer von Röslerstamm, 1842), *U. nebulalis* (Hübner, 1796), *U. rhododendronalis* (Duponchel, 1834), *U. uliginosalis* (Stephens, 1834). Furthermore, *U. uralica* Slamka, 2013 exhibits the group-specific apomorphies (see below) and is here added to the *U. alpinalis* group.

The *U. alpinalis* species group is characterised by a homogenous wing colouration with an inconspicuous maculation. Species of this group exhibit sexual dimorphism, with females having shorter, more acute forewings and the dorsal side of the hindwings being usually darker than in males. Furthermore, the *U. alpinalis* group is distinguished from other *Udea* species groups by the presence of a sclerotised protrusion of variable shape on the posterior phallus apodeme. The species inhabit montane regions, and the larvae exhibit a range of feeding habits from monophagy to polyphagy on a variety of herbaceous plants (Lhomme 1935; Huemer and Tarmann 1989; Slamka 2013).

Several authors suspect that the actual species diversity in the *U. alpinalis* group in Europe is higher than formal descriptions in the literature indicate, specifically in relation to *U. austriacalis* and *U. rhododendronalis*. This suspicion is based on small differences in genitalia structure and wing maculation (Zerny 1914, Filipjev 1925, Leraut 1996, Slamka 2013). For *U. alpinalis*, Galvagni (1933), Weber (1945), and Panigaj and Kulfan (2012) found considerable variability in the forewing maculation. In preliminary COI Barcode cluster analyses, several specimens that have been identified as *U. uliginosalis* based on wing maculation clustered with *U. alpinalis*, raising questions about the correct species identification.

In this study, these taxonomic suspicions are addressed through the analysis of morphological as well as mitochondrial and nuclear genetic data. We present, investigate, and, where possible, explain this unsettled question of intraspecific diversity of *U. rhododendronalis*, *U. austriacalis* and the *U. uliginosalis*-*U. alpinalis* species pair.

## Materials and methods

The study is based on adult specimens of *Udea alpinalis*, *U. austriacalis*, *U. cretacea, U. rhododendronalis*, and *U. uliginosalis*, collected at different localities in Europe and Central Asia. The genetic dataset was complemented with sequences of *U. bourgognealis*, *U. carniolica*, *U. murinalis*, and *U. nebulalis*. *Udea ruckdescheli* Mally, Segerer & Nuss, 2016 of the *U. numeralis* species group (sensu Mally and Nuss 2011) served as outgroup. The morphologically investigated material is summarised in the ‘material examined’ sections of the respective species in the taxonomic results, the genetic data are summarised in Table 2.

Molecular data from three different genes were used for the dataset: the 5’ half of the mitochondrial Cytochrome c oxidase subunit 1 (COI) gene (the “DNA Barcode”), 657 base pairs (bp) in length, the 5’ part of the nuclear Elongation factor 1-alpha (EF1a) gene (780 bp), and the nuclear Wingless gene (372 bp). In addition, a screening for molecular traces of *Wolbachia* infections was done by amplifying the bacterial markers *Wolbachia* surface protein (wsp), aspartyl/glutamyl-tRNA(Gln) amidotransferase subunit B (gatB) and Filamenting temperature-sensitive mutant Z (ftsZ).

COI Barcode sequences and specimen data for the *Udea* species of interest were obtained from ongoing Barcoding projects of PH and MN on the Barcoding of Life Database (BOLD, www.boldsystems.com), Version 4. The DNA lab protocols at the Canadian Centre for DNA Barcoding (CCDB) are available at http://www.ibolproject.org/resources.php. Barcodes with less than 500 bp were excluded, and public records retrieved from NCBI GenBank were included. In addition, DNA Barcode sequences were obtained for several specimens through PCR and sequencing in the DNA labs of the Senckenberg Natural History Collections Dresden, Germany (SNSD) and the Institute of Biology at the University of Bergen, Norway (UiB).

For DNA lab protocols at SNSD see Mally and Nuss (2011). The DNA lab protocols at UiB are as follows: The abdomen was detached from the dried specimen and DNA was extracted using the DNeasy Blood & Tissue kit (Qiagen) according to the manufacturer’s protocol. Gene sequences were amplified in 25 µl reactions from 2 µl DNA extract using 400 nM of each primer, 800 µM dNTP mix, 2.5 µl Taq buffer (incl. MgCl_2_), 0.75u TaKaRa Ex Taq DNA Polymerase and distilled water added up to 25 µl in total per reaction. COI primers were HybLCO (forward) and HybNancy (reverse) (Folmer et al. 1994, Wahlberg and Wheat 2008), EF1a primers were HybOscar-6143 (forward) and Bosie-6144 (reverse) (Wahlberg and Wheat 2008, Haines and Rubinoff 2012), and Wingless primers were HybLepWg1 (forward) and HybLepWg2 (reverse) (Wahlberg and Wheat 2008). Those primers contained the universal primer tail pair T7/T3 (‘Hyb’ in the primer names; Wahlberg and Wheat 2008), which were used for sequencing. The wsp gene was amplified using the primers WspecF and WspecR (Werren and Windsor 2000). In cases of lacking amplification success the internal primers INTF1 and INTR2 (Sakamoto et al. 2006) were used. The genes gatB and ftsZ were amplified using the primers of Baldo et al. (2006) in combination with the universal forward (T7 promoter) and reverse (T3) tails (Wahlberg and Wheat 2008).

The PCR programme for COI was: initial phase at 95 °C for 5 min, 38 cycles with 95 °C for 30 s, 50 °C for 30 s and 72 °C for 60 s, final phase at 72 °C for 10min and cooling at 8 °C. For EF1a and Wingless a touchdown PCR was performed: 24 cycles at 95 °C for 30 s, 55 °C with -0.4 °C/cycle for 30 s and 72 °C for 60 s +2s/cycle, then 12 cycles at 95 °C for 30 s, 45 °C for 30 s and 72 °C for 120 s +3 s/cycle, final phase at 72 °C for 10min and cooling at 8 °C. The PCR protocol of Sakamoto et al. (2006) was used for For wsp, and the protocol of Baldo et al. (2006) for gatB and ftsZ.

PCR results were examined via gel electrophoresis on a 1 % agarose gel and GelRed as dye agent. Successful PCR samples were cleaned with ExoSAP and subsequently amplified in Sanger-sequencing PCR reactions for both primers using the BigDye kit and this setup: 0.5–3.0 µl of PCR sample (depending on the sample’s band thickness on the agarose gel), 160 nM primer, 1 µl buffer, 0.5 µl BigDye, and adding up distilled water to 10 µl in total per reaction. Sequencing was conducted at the sequencing facility of UiB, Dept. of Molecular Biology. PCR and sequencing PCR were performed on a Bio-Rad 1000 thermal cycler; ExoSAP clean-up was done on a MJ Research PTC-200 thermal cycler.

The three gene datasets (COI, Wingless, EF1a) were aligned with PhyDE 0.9971 (Müller et al. 2008) and analysed individually with raxmlGUI v. 1.5b2 (Stamatakis 2006; Silvestro and Michalak 2012), using a Maximum Likelihood (ML) search under the GTRGAMMA model (Rodriguez et al. 1990) and with a thorough bootstrap of 1,000 Bootstrap replicates. Phylograms were edited in TreeGraph version 2.13.0-748 beta (Stöver and Müller 2010). The corresponding alleles and supergroups of successful *Wolbachia* sequences were sought in the BIGSdb database (Jolley and Maiden 2010).

Dissection of genitalia was performed according to Robinson (1976), with modifications. In order to preserve the tympanal organ, the abdomen was cut open longitudinally along one pleural membrane, detached from the genitalia, cleaned, and embedded under a separate cover slip next to the cover slip with the genitalia. Morphological structures were investigated using a Leica M125 stereomicroscope. Photographic documentation of imagines was done with a Canon EOS 60D in combination with a Canon EF 100mm 1:2,8 Macrolens and Canon EOS Utility Version 2.10.2.0 on a Windows PC. A Leica CTR6000 microscope in combination with a Leica DFC420 camera and Leica Application Suite programme (Version 3.8.0) on a Windows PC was used for documentation of the genitalia. Images were edited in GIMP 2.8.6. The distribution maps were generated with DIVA-GIS version 7.5.0.0 (Hijmans et al. 2001) and SRTM 90 m digital elevation data (Jarvis et al. 2008).

### Abbreviations

The abbreviations of the insect collections follow Evenhuis (2017).

**EF1a** Elongation Factor 1-alpha

**ftsZ** Filamenting temperature-sensitive mutant Z

**gatB** aspartyl/glutamyl-tRNA(Gln) amidotransferase, subunit B

**GTR** General time reversible substitution model (see Rodriguez et al. 1990)

**ML** Maximum Likelihood

**MLST** Multilocus sequence typing

**MTD** Senckenberg Natural History Collections, Museum of Zoology Dresden

**NHMUK** Natural History Museum London, UK

**NHMW** Natural History Museum Vienna, Austria

**SMNK** State Museum of Natural History Karlsruhe, Germany

**TLMF** Tiroler Landesmuseum Ferdinandeum, Innsbruck, Austria

**wsp** *Wolbachia* surface protein

**ZMBN** Zoological Museum, University of Bergen, Norway

**ZMHB** Zoological Museum, Humboldt University Berlin, Germany

**ZMUC** Zoological Museum, University Copenhagen, Denmark

**ZSM** Zoological State Collections Munich, Germany

## Results

### Molecular results

In total, genetic data were analysed for 80 specimens (see Table 2) of the *U. alpinalis* group, specifically for *U. alpinalis*, *U. austriacalis*, *U. rhododendronalis* and *U. uliginosalis*. COI data were available for 77 specimens, EF1a for 31 specimens, and wingless for 29 specimens. The low coverage of nuclear genetic data is due to the age of most specimens, with the nuclear genome being too fragmented to be sequenced with the classical Sanger approach.

**Table 1.** Ratios of length versus breadth of the main signum of female genitalia in selected species of the *Udea alpinalis* species group.

Ratio length to breadth of main signum	*Udea austriacalis* (n = 8)	*U. donzelalis* (n = 7)	*U. altaica* (n = 4)
**minimum**	3.44	4.06	3.09
**maximum**	4.375	5.08	3.44
**Average**	**3.85**	**4.56**	**3.25**
**standard deviation**	0.36	0.41	0.15

**Table 2.** Origin and gene sequence data of the genetically investigated *Udea* material.

species	Origin	BOLD sample no.	DNA extraction no.	COI accession no.	EF1a accession no.	wingless accession no.
***U. austriacalis***	Austria, Carinthia	TLMF Lep 00837	ZMBN Lep419	HQ968213	MG523969	MG523989
	Macedonia, Mavrovo Nat. Park	TLMF Lep 05069	ZMBN Lep420	KX042511	–	MG523990
	Italy, South Tyrol	BC MTD 00758	ZMBN Lep421	JF852277	MG523970	MG523991
	Italy, Belluno	TLMF Lep 00570	ZMBN Lep422	HM381412	MG523971	MG523992
	Italy, Cuneo	TLMF Lep 00971	ZMBN Lep423	HM381536	MG523972	MG523993
	Italy, Piedmont	BC MTD 01617	ZMBN Lep424	MG191924	–	–
	Italy, South Tyrol	–	MTD Lep238	JF497036	MG523942	–
	Italy, South Tyrol	–	MTDLep292	–	MH078064	JF497077
	Italy, Cuneo	–	MTD Lep960	MG523926	MG523946	–
	France, Alpes-Maritimes	TLMF Lep 00988	MTD Lep961	HQ968447	MG523947	–
	France, Basses-Alpes	–	MTD Lep962	MG523927	–	–
	Italy, Cuneo	TLMF Lep 00527	–	HM381372	–	–
	France, Alpes-Maritimes	TLMF Lep 00633	–	HM381456	–	–
	Austria, Carinthia	TLMF Lep 00838	–	HQ968214	–	–
	Italy, Cuneo	TLMF Lep 00970	–	HM381535	–	–
	France, Provence-Cote d’Azur	TLMF Lep 00987	–	HM381552	–	–
	Macedonia, Mavrovo Nat. Park	TLMF Lep 05068	–	KX042766	–	–
***U. donzelalis***	Andorra	–	ZMBN Lep084	MG523936	MG523962	MG523983
	Andorra	–	ZMBN Lep085	MG523937	MG523963	MG523984
	France, Cantal	–	ZMBN Lep090	MG523938	–	MG523985
	France, Cantal	–	ZMBN Lep091	MG523939	MG523964	MG523986
	Spain, Huesca	TLMF Lep 20010	–	MG191926	–	–
***U. cretacea***	Russia, Kabardino-Balkaria	BC MTD Lep 01612	–	MG191928	–	–
***U. rhododendronalis***	Macedonia, Mavrovo Nat. Park	TLMF Lep 05086	ZMBN Lep073	KX042769	–	MG523974
	Andorra	–	ZMBN Lep074	MG523933	MG523953	MG523975
	Spain, Cantabria	–	ZMBN Lep075	MG523934	–	–
	Spain, Cantabria	–	ZMBN Lep076	MG523935	MG523954	–
	France, Alpes-Maritimes	–	ZMBN Lep413	MG523940	–	–
	Austria, Styria	TLMF Lep 00900	ZMBN Lep414	HQ968270	MG523966	MG523987
	Austria, Vorarlberg	TLMF Lep 09148	ZMBN Lep415	KP253729	MG523967	–
	Italy, South Tyrol	TLMF Lep 09218	ZMBN Lep416	MG191932	–	–
	Italy, Cuneo	TLMF Lep 00972	ZMBN Lep417	HM381537	MG523968	MG523988
	Spain, Lerida	–	ZMBN Lep418	MG523941	–	–
	Austria, East Tyrol	BC MTD Lep 00768	MTD Lep242	JF852287	MG523944	MG523973
	Italy, Cuneo	–	MTD Lep288	MG523923	MG551293	JF497100
	Spain, Cantabria	–	MTD Lep1390	MG523928	MG523948	–
	Andorra	–	MTD Lep1391	MG523929	MG523949	–
	Austria, Styria	TLMF Lep 00899	–	HM381472	–	–
	Macedonia, Mavrovo Nat. Park	TLMF Lep 05082	–	KX042320	–	–
	France, Midi-Pyrenees	TLMF Lep 05660	–	MG191933	–	–
***U. alpinalis***	Austria, Carinthia	TLMF Lep 00535	ZMBN Lep082	HM381379	MG523960	MG523981
	Austria, Styria	TLMF Lep 00897	ZMBN Lep083	HM381470	MG523961	MG523982
	Switzerland, Valais	–	MTD Lep258	JF497035	–	–
	Italy, South Tyrol	–	MTD Lep295	MG523924	MG523945	JF497076
	Italy, South Tyrol	BC MTD Lep 00754	–	JF852273	–	–
	Austria, Tyrol	BC MTD Lep 00755	–	JF852274	–	–
	Austria, Carinthia	TLMF Lep 00534	–	HM381378	–	–
	Italy, Belluno	TLMF Lep 00568	–	HM381410	–	–
	Austria, Styria	TLMF Lep 00897	–	HM381470	–	–
	Austria, Styria	TLMF Lep 00898	–	HM381471	–	–
	Austria, Vorarlberg	TLMF Lep 08390	–	KP253445	–	–
***U. uliginosalis***	Macedonia, Mavrovo Nat. Park	TLMF Lep 05088	ZMBN Lep079	KX042480	MG523957	MG523978
	Austria, Carinthia	TLMF Lep 00823	ZMBN Lep080	HQ968199	MG523958	MG523979
	France, Alpes-Maritimes	TLMF Lep 00635	ZMBN Lep081	HM426003	MG523959	MG523980
	Italy, South Tyrol	–	MTD Lep239	JF497067	MG523943	–
	Slovenia, Bovec	BC MTD Lep 521	–	HQ960224	–	–
	Italy, South Tyrol	BC MTD Lep 00756	–	JF852275	–	–
	Austria, Carinthia	TLMF Lep 00824	–	HQ968200	–	–
	Austria, Vorarlberg	TLMF Lep 00973	–	HM381538	–	–
	Austria, Carinthia	TLMF Lep 01021	–	HM381585	–	–
	France, Provence-Cote d’Azur	TLMF Lep 01022	–	HM381586	–	–
	Italy, Udine	TLMF Lep 01461	–	HQ968677	–	–
	Slovenia	TLMF Lep 01703	–	HQ968907	–	–
	Slovenia	TLMF Lep 01704	–	HQ968908	–	–
	Macedonia, Mavrovo Nat. Park	TLMF Lep 05087	–	KX042181	–	–
***U. uliginosalis* x *alpinalis***	Austria, Tyrol	–	MTD Lep297	MG523925	–	JF497102
	Austria, Styria	TLMF Lep 00896	ZMBN Lep077	HQ968269	MG523955	MG523976
	Italy, Belluno	TLMF Lep 00577	ZMBN Lep078	HM381419	MG523956	MG523977
	Austria, Tyrol	BC MTD Lep 00757	MTD Lep1582	JF852276	MG523950	–
	Austria, Tyrol	–	MTD Lep1583	MG523930	–	–
	Austria, Tyrol	–	MTD Lep1584	MG523931	MG523951	–
	Austria, Tyrol	–	MTD Lep1585	MG523932	MG523952	–
***U. juldusalis***	Kyrgyzstan, Ysyk-Kol	BC MTD Lep 522	–	HQ960225	–	–
	Kazakhstan, Almaty Region	BC MTD Lep 523	–	HQ960226	–	–
***U. murinalis***	Austria, Vorarlberg	–	MTD Lep287	JF497057	–	JF497094
***U. nebulalis***	Austria, East Tyrol	–	MTD Lep251	JF497057	–	–
	Austria, East Tyrol	–	MTD Lep293	–	–	JF497095
***U. bourgognealis***	France, Alpes-Maritimes	–	MTD Lep350	JF497038	–	–
	France, Alpes-Maritimes	–	MTD Lep351	–	–	JF497078
***U. carniolica***	Italy, South Tyrol	–	MTD Lep289	JF497039	–	JF497079
***U. ruckdescheli* (outgroup)**	Greece, Crete	–	ZMBN Lep150	LT595885	MG523965	LT595888

**Table 3.** COI Barcode p-distances of the systematically investigated *Udea* populations.

DNA Barcode group	n	range of intraspec. p-distance [%]	average intraspec. p-distance [%]	nearest neighbour(s) (NN)	range of interspec. p-distance to NN [%]	average interpec. p-distance to NN [%]
*U. austriacalis* (C- & E-Alps, Macedonia)	7	0–0.95	0.45	*U. austriacalis* (Maritime Alps)	2.38–3.33	2.86
*U. austriacalis* (Maritime Alps)	9	0	0	*U. austriacalis* (C- & E-Alps, Macedonia) / *U. cretacea*	2.38–3.33 / 2.38	2.86 / 2.38
*U. donzelalis*	5	0	0	*U. cretacea*	4.29	4.29
*U. cretacea*	1	n/a	n/a	*U. austriacalis* (Maritime Alps)	2.38	2.38
*U. rhododendronalis* (Pyrenees)	7	0–0.48	0.27	*U. rhododendronalis* (Alps)	2.38–3.33	3.07
*U. rhododendronalis* (Alps)	8	0–0.48	0.12	*U. rhododendronalis* (Macedonia)	0.95–1.43	1.01
*U. rhododendronalis* (Macedonia)	2	0	0	*U. rhododendronalis* (Alps)	0.95–1.43	1.01
*U. juldusalis*	2	0	0	*U. uliginosalis*	2.86–4.29	3.30
*U. uliginosalis*	14	0–2.86	1.03	*U. alpinalis*	1.43–4.29	2.64
*U. alpinalis*	11	0–2.86	0.99	*U. uliginosalis* / *U. alpinalis* x *uliginosalis* hybrids	1.43–4.29 / 0.95–3.33	2.64 / 2.76
*U. alpinalis x uliginosalis* hybrids	7	0–2.38	1.11	*U. alpinalis*	0.95–3.33	2.71

**Figure 1.**
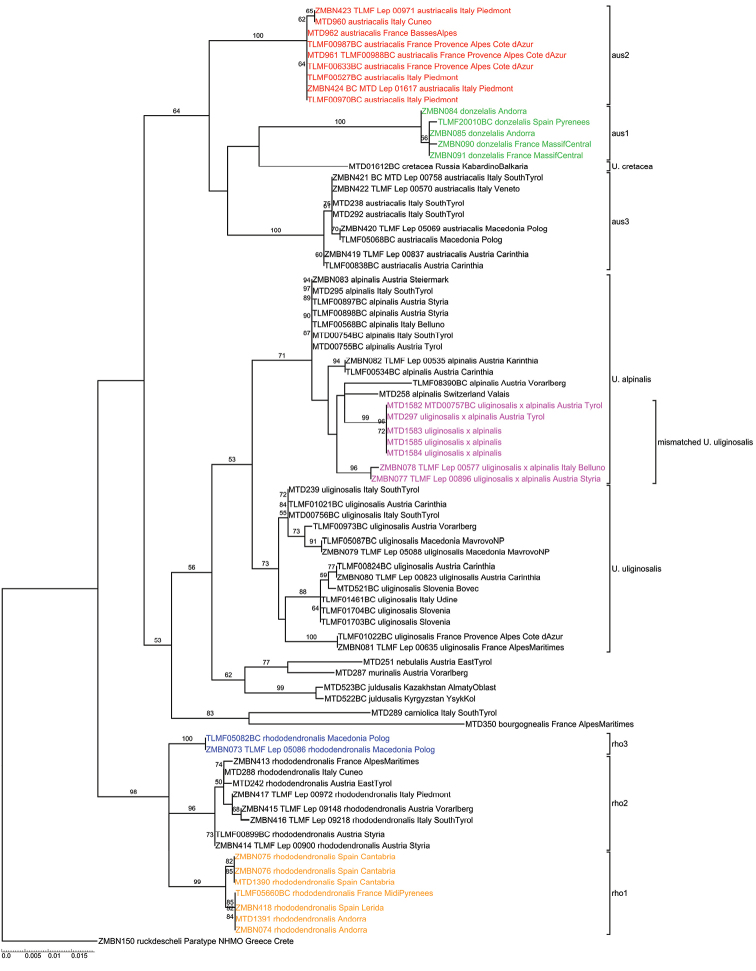
Maximum Likelihood analysis of COI Barcode data of the *Udea alpinalis* species group. Numbers on branches represent bootstrap values of ≥ 50 % inferred from 1,000 replicates, scale bar represents substitutions per site.

Analysis of COI resulted in a gene tree (Fig. 1) with several deep intraspecific, geographically coherent clades for *U. austriacalis* and *U. rhododendronalis*. *Udea austriacalis* splits into three groups: (aus1) Pyrenees and the French Massif Central (green clade in Fig. 1), (aus2) the French and Italian Maritime Alps (red clade), and (aus3) the Central and Eastern Alps as well as the Balkan Mountains (black clade). A fourth clade within *U. austriacalis* is represented by a single specimen of *U. cretacea*, indicating that *U. austriacalis* is non-monophyletic. *Udea rhododendronalis* splits into three COI clades: (rho1) Pyrenees (orange clade in Fig. 1); (rho2) Alps (black clade); (rho3) Southern Balkan Mountains (blue clade). Seven specimens of *U. uliginosalis* (marked in pink in Fig. 1) group with the *U. alpinalis* clade instead of with the other *U. uliginosalis* specimens. These seven specimens originate from three different localities in the Central Alps (see Tab. 2); the specimens collected at Hahntennjoch (Tyrol, Austria) group together, while the specimen from Styria (Austria) groups with the specimen from Belluno (Italy). All seven mismatched *U. uliginosalis* specimens are males. The two specimens identified as *U. juldusalis* are sister to the clade *U nebulalis* + *U. murinalis*.

**Figures 2–3.**
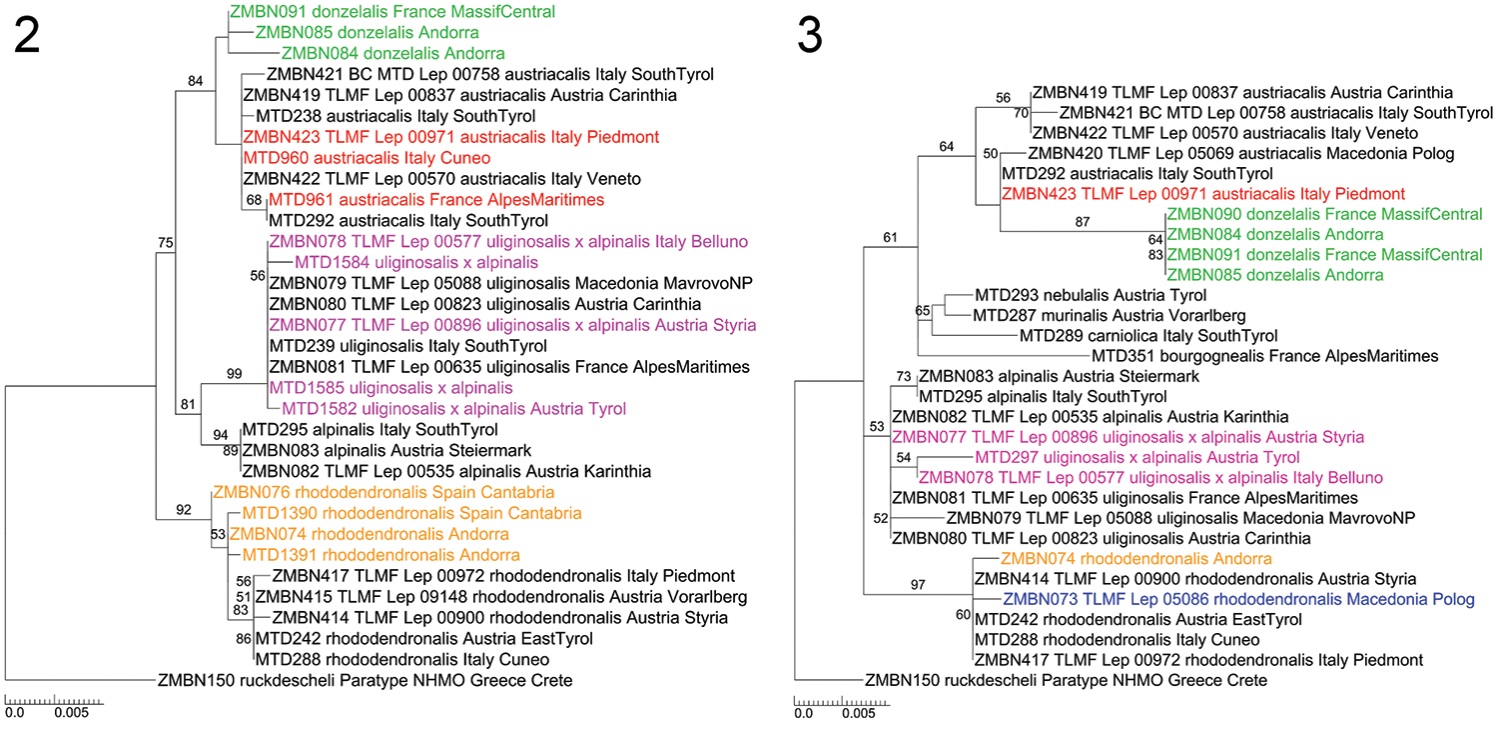
Maximum Likelihood analysis of EF1a (**2**) and wingless (**3**) data of the *Udea alpinalis* species group. Numbers on branches represent bootstrap values of ≥ 50% inferred from 1,000 replicates. Note that the taxon set is not identical for the two analyses, scale bars represent substitutions per site.

In the ML analysis of EF1a under the GTRGAMMA model, values for alpha were often above 10, and the EF1a dataset was analysed with the GTR model instead. In the resulting EF1a gene tree (Fig. 2), two *U. austriacalis* clades are found: one clade containing all three successfully sequenced specimens from group aus1, originating from the Pyrenees and the Massif Central (green clade), and one clade containing specimens from groups aus2 and aus3 (*U. austriacalis* samples marked in red and black). In comparison to the COI results, *U. rhododendronalis* does not group into distinct clades, and specimens from groups rho1 and rho2 form a single clade instead; no specimen from the group rho3 could be sequenced successfully. All specimens of *U. uliginosalis*, including those that group with *U. alpinalis* in the COI gene tree, form a monophyletic clade that is sister to the monophyletic *U. alpinalis* clade.

In the wingless gene tree (Fig. 3), specimens of the *U. austriacalis* clade aus1 form a monophylum on a long branch that is nested in the clade containing specimens from group aus3 and the single successfully amplified specimen from group aus2. *Udea rhododendronalis* specimens of all three COI Barcode groups (rho1–3) form a single monophyletic clade. *Udea alpinalis* and *U. uliginosalis* are not distinguished in two clades but form a common clade instead, including the *U. uliginosalis* specimens with the COI Barcode mismatch.

The three bacterial markers wsp, gatB, and ftsZ were used in order to screen for *Wolbachia* in a number of *Udea* specimens. Sequencing was successful in only two of the twelve tested specimens: a specimen of *U. fulvalis* from Crete collected 5.44 years before DNA extraction, and a specimen of *U. olivalis* from Armenia collected 4.26 years before DNA extraction. For the other ten tested specimens, PCR amplification produced a band in some cases, but sequencing failed.

For the two successful samples, wsp sequences produced no match in BIGSdb (Jolley and Maiden 2010). For the specimen of *U. fulvalis* (voucher ZMBN Lep125), the gatB sequence had the closest match with gatB allele 196, found in MLST profile 306; the ftsZ sequence had the closest match with ftsZ allele 36, present in 12 MLST profiles (41, 42, 109, 145, 146, 150, 151, 156, 157, 235, 305, 374). Both gatB and ftsZ place the *Wolbachia* strain from the *U. fulvalis* specimen into supergroup B. For the specimen of *U. olivalis* (voucher ZMBN Lep156), gatB had the closest match with gatB allele 7, present in seven MLST profiles (19, 112, 118, 261, 266, 268, 272); ftsZ resulted in an exact match with ftsZ allele 3, present in 31 MLST profiles (10, 13, 14, 17, 19, 24, 25, 54, 80, 83, 89, 91, 92, 107, 112, 118, 122, 123, 127, 132, 133, 165, 199, 234, 330, 404, 432, 434, 449, 451, 454). Both gatB and ftsZ place the *Wolbachia* strain from the *U. olivalis* specimen into supergroup A. The gatB and ftsZ sequences obtained from *U. fulvalis* and *U. olivalis* can be obtained from RM.

### Taxonomy

Based on morphological, genetic, and biogeographical data, the following changes in the taxonomy of the species of the *U. alpinalis* species group are proposed, and redescriptions are provided.

#### Udea austriacalis

Taxon classificationAnimaliaLepidopteraCrambidae

(Herrich-Schäffer, 1851) stat. rev.

Figs 1 , 2–3 , 4 , 7–10 , 23–28 , 45

Botys austriacalis Herrich-Schäffer, 1851: 288 [1851 – binominal], pl. 20 fig. 142 [1849 - uninominal].
= Botys nitidalis Heinemann, 1865: 83.
= Botys sororialis Heyden, 1860: 93.

##### Type locality.

Austria, Carinthia, Hohe Tauern, Grossglockner.

##### Material examined.

**Central Alps and Balkan clade (BOLD BIN AAD2364; aus3 in Fig. 1): AUSTRIA.** 1♂ “Austria merid., Kärnten | Dellach im Drautal | Zollnertörl | 13°04'24"E, 46°35'58"N | 1830 m, 1.7.2009 | leg. Huemer | TLMF 2009-138”, [pale green label] “BC TLMF Lep 00837”, [salmon-pink label] “DNA voucher | Lepidoptera | ZMBN 2016 | [transverse] no. 419”, Mally prep. no. 1041 (TLMF); 1♂ same data but without DNA voucher label and with [pale green label] “BC TLMF Lep 00838”, Mally prep. no. 1109 (TLMF); 1♂ “[handwritten] Vent | Oetztal | [handwritten] 19.VII1942 | E. Möbius”, “Coll. STARKE / Bautzen | Ankauf 1953 | Übernahme 1969”, Mally prep. no. 9 (MTD); 1♂ “Teriolis | Ötztal [handwritten] 2000 m | Vent [handwritten] 31/7 1926” (NHMW); **SWITZERLAND.** 1♂ “Lukmanierpass | W. Heinitz | 1910”; “zu prüfen! | Pionea nebulalis | Pyrausta austriacalis | sororialis”, “20069”, “Coll. W. Heinitz | Ankauf 1950”, Mally prep. no. 417 (MTD); 1♂ “Fusio, | [handwritten] 20. July, 1917, | (K.J. & N.C.R.)”, [handwritten] Pyrausta | austriacalis | ♂ H.-S.”, “Rothschild | Bequest | B.M.1939-1.”, Mally prep. no. 1092 (NHMUK); 1♀ “[handwritten] Pontresi- | na. 61 10/4”, “♀”, “ 21513”, “Coll. W. Heinitz | Ankauf 1950”, Mally prep. no. 10 (MTD); 1♀ [handwritten] “Pontresina | Switz | 12.VII.1965 | [printed] S.N.A.JACOBS.”, “Brit. Mus. | 197 [handwritten]2 305”, Mally prep. no. 1091 (NHMUK); **ITALY.** 1♂ “Italien, Prov. Südtirol | Kastelruth, Saltner Hütte SE | 1870 m, 17.6.2007 | leg. Huemer | TLMF 2008-009”, “Udea austriacalis | det. P. Huemer 2008”, [orange label] “DNA voucher | Lepidoptera | M. Nuss 2007 | [transverse] no. 238”, Mally prep. no. 69 (TLMF); 1♂ “Italy, Südtirol, Sellagruppe | Sela de Culac, 2018m, | Wirtschaftswiese, Tagfang | 05.08.2008, leg. Nuss et al.”, [pale green label] “BC MTD 00758”, [salmon-pink label] “DNA voucher | Lepidoptera | ZMBN 2016 | [transverse] no. 421”, Mally prep. no. 1043 (MTD); 1♂ “Italien, Prov. Belluno | Passo di Valparola E - Passo | Falzarego | 12°0'25,1"E, 46°31'20,6"N | 2150 m, 20.-21.7.2009 | leg. Huemer | TLMF 2009-138”, [pale green label] “BC TLMF Lep 00570”, [salmon-pink label] “DNA voucher | Lepidoptera | ZMBN 2016 | [transverse] no. 422”, Mally prep. no. 1044 (TLMF); 1♂ [handwritten] “Prad. Alp. | b.Trafoi | Juli 871.” (NHMW); 1♂ [handwritten] “Rognhofr | Stilfser | Joch | 1871.” (NHMW); 1♂ “Sella-Joch, | Rbl. VII” (NHMW); 1♀ “Sellajoch | W. Heinitz | 1912”, “♂”, [handwritten] “25736”, “Coll. W. Heinitz | Ankauf 1950”, Mally prep. no. 50 (MTD); 1♀ “Italy, Südtirol, Sellagruppe | Sela de Culac, 2018m, | Wirtschaftswiese, Tagfang | 05.08.2008, leg. Nuss et al.”, [orange label] “DNA voucher | Lepidoptera | M. Nuss 2007 | [transverse] no. 292”, Mally prep. no. 114 (MTD); **ALBANIA and MACEDONIA.** 1♂ “Macedonia, NP Mavrovo | Korab, Korabska jezero, | Kobilino pole, 2080–2180 m | 20°34'55"E, 41°46'42"N | 28.7.–1.8.2011 | leg. Huemer & Tarmann”, [pale green label] “BC TLMF Lep 05069”, [salmon-pink label] “DNA voucher | Lepidoptera | ZMBN 2016 | [transverse] no. 420”, Mally prep. no. 1042 (TLMF); 1♂ 1♀ “Alban.Exp.1918 | Korab,23–31.VII”, Mally prep. no. 1096 (♀) (NHMW); **BULGARIA.** 2♂ “Ende | Juli”, “Bulgarien | Rebel ’02. | [handwritten] Rila 1300 [underlined] m” (NHMW); 1♂ [handwritten] “Rila | c.1800 m | 25.VII 02” (NHMW); **RUSSIA.** 1♂ “Kaukasus | [handwritten] Dombai | Tsihutsihur- | Tal leg. Alberti; [Unterseite] 2300 m | 5.7.1968”, [green label, handwritten] “1 | Amsel”, [handwritten] austriacalis”, Mally prep. no. 205 (ZSM);

##### Maritime Alps Barcode clade

**(BOLD BIN AAD2363; aus2 in Fig. 1): FRANCE.** 1♂ “Frankreich, Alpes-Maritimes | PN Mercantour | N Col de la Cayolle | Col de la Boucharde N | 6°44'36"E, 44°17'00"N | 1930–1950m, 26.7.2009 | leg. Huemer”, [pale green label] “BC TLMF Lep 00988”, [orange label] “DNA voucher | Lepidoptera | Mally 2011 | [transverse] no. LEP961”, Mally prep. no. 449 (TLMF); 1♂ [handwritten] “NÉVACHE | HAUTES-ALPES | July 29-Aug.16, 1924 | Wm Fassnidge.”, “Brit. Mus. | 197[handwritten]2 305”, [folded label] “2059.austriacalis [handwritten] 123 | Herrich-Schaffer.” (NHMUK); 1♂ “Valloire, Savoie, | 11. July 1910. | (W. R. & K. J.)”, “Rothschild | Bequest | B.M.1939-1.”, Mally prep. no. 1105 (NHMUK); 1♂ “La Grave, Hautes Alpes, | 1500–1800 m. | 21. July 1908. | (W. R. & K. J.)”, “Rothschild | Bequest | 1939-1.” (NHMUK); 1♂ “Le Lautaret, | Hautes Alpes, | 2000–2300 m., | 4. August 1908. | (W. R. & K. J.)”, “Rothschild | Bequest | 1939-1.” (NHMUK); 1♂ “Frankreich, Alpes-Maritimes | PN Mercantour | N Col de la Cayolle | Col de la Boucharde N | 6°44'36"E, 44°17'00"N | 1930–1950 m, 26.7.2009 | leg. Huemer”, [turquoise label] “BC TLMF Lep 00633” (TLMF); 1♂ “Frankreich, Alpes-Maritimes | PN Mercantour | N Col de la Cayolle | Col de la Boucharde N | 6°44'36"E, 44°17'00"N | 1930–1950 m, 26.7.2009 | leg. Huemer”, [turquoise label] “BC TLMF Lep 00987” (TLMF); 1♂ “MAURIN.B-ALPES | [handwritten] 25 8 1932 | W.FASSNIDGE.”, “Brit. Mus. | 197[handwritten]2 305”, Mally prep. no. 1106 (NHMUK); 1♀ same data but “1 8 1932”, Mally prep. no. 1101 (NHMUK); 1♀ “Frankreich, Dep. Basses Alpes | SW Castel de Restfond | Ste. De Caire Brun N | 2420m, 25.–26.7.1990 | leg. Huemer & Tarmann”, [orange label] “DNA voucher | Lepidoptera | Mally 2011 | [transverse] no. 962”, Mally prep. no. 450 (TLMF); **ITALY.** 1♂ “Italien, Prov. Cuneo | Alpi Cozie, Demonte NW | 44°23'04"N 7°6'23"E | 2.8.2010 | leg. Huemer | TLMF 2011-010”, [orange label] “DNA voucher | Lepidoptera | Mally 2011 | [transverse] no. 960”, Mally prep. no. 448 (TLMF); 1♂ “Italy, Prov. Piemonte, | Colle di Lombarda, | 2300 m, 25.vii.2006, | Peder Skou leg.”, [yellow label] “Coll. ZMUC | Copenhagen, DK”, [pale green label] “BC MTD 01617”, [salmon-pink label] “DNA voucher | Lepidoptera | ZMBN 2016 | [transverse] no. 424”, Mally prep. no. 1046 (ZMUC); 1♂ “Italien, Prov. Cuneo | Alpi Cozie, Demonte NW | Gias Valcavera | 7°8,2'E, 44°22,6'N | 2050 m, 23.7.2009 | leg. Huemer | TLMF 2009-138”, [turquoise label] “BC TLMF Lep 00971”, [salmon-pink label] “DNA voucher | Lepidoptera | ZMBN 2016 | [transverse] no. 423”, Mally prep. no. 1045 (TLMF); 1♀ “Piemont,Colle di | Sestrières,18–2100m | 23.–31.VII.’37,Zerny”, Mally prep. no. 1097 (NHMW).

##### Diagnosis.

Outer side of labial palps’ 2^nd^ and 3^rd^ palpomeres pronouncedly darker than the rest of the labial palps; in *U. donzelalis*, labial palps’ 2^nd^ and 3^rd^ palpomeres on outside barely darker than rest of labial palps. Maculation of forewing usually less pronounced than in *U. donzelalis*, the apical brown streak often narrower; hindwing in females dorsally evenly dark brown, whereas in *U. donzelalis* the inner area is greyish cream-white, contrasted by a darker outer band. In the male genitalia, the fibula of *U. austriacalis* is generally a bit narrower and more evenly broad from the base to the subapex; in *U. donzelalis* the fibula is somewhat broader and elongate triangular. In the female genitalia, the signum of *U. austriacalis* is 3.4–4.4 times as long as broad (n = 8), whereas in *U. donzelalis* the signum is on average narrower, being 4.1–5.1 times as long as broad (n = 6) (Tab. 1). *Udea austriacalis* can furthermore be distinguished by the COI Barcode from all other sequenced *Udea* species; the two allopatric DNA barcode clades of *U. austriacalis*, the first confined to the Maritime Alps and the second from other parts of the Alps, the Balkan Mountains and the Caucasus, do not differ from each other morphologically and in the nuclear genes wingless and EF1-alpha, so that they are considered conspecific. The two COI Barcode clades are the nearest neighbours to each other, and they differ by a minimum of 2.38 % p-distance of nucleotide divergence from each other; the Maritime Alps clade also has *U. cretacea* as nearest neighbour with 2.38% p-distance of minimum nucleotide divergence (Tab. 3).

##### Redescription.

*Head*. Frons and vertex covered with beige scales; frons evenly convex, covered with beige to light brown scales; labial palps projecting forward, third segment pointed, palps covered with beige scales, outer sides of labial palps’ second and third segment light to dark brown; maxillary palps approximately one quarter as long as labial palps, with beige apical scale tuft; compound eyes hemispherical; proboscis well developed, its base covered in pale beige and dark brown scales; antennae filiform, posterior side covered in pale beige scales, anterior side in males densely covered with cilia shorter than the basal antennal radius, shorter still in females, antennal length approx. 50–60 % of forewing length in males, approx. 70 % in females; ocellus posterior to antenna base.

*Thorax*. Cream-white, with collar and anterior part of tegulae scales more caramel-coloured; legs cream-white except for dark brown inner side of fore- and midlegs as well as distal half of hindlegs; tibial spurs on fore-/mid-/hindleg 0/2/4, as in other species of the genus; midleg outer spur ca. 2/3 length of inner spur; hindleg outer spurs ca. 2/3 length of inner spurs.

*Wings*. Forewing length 11–13 mm in males, 8–10 mm in females. Males and females with one frenulum bristle. Females with more acute apex due to the straight costa (distally curved in males). Forewing ground colour glossy cream-white to beige, with maculation more or less prominent: basal two thirds of costa with light brown streak, distal discoidal stigma a diffuse brownish dot, postmedial line brownish, arching around distal discoidal stigma, then turning basad until below distal discoidal stigma, sharply arching back outwards (the typical “*Udea* loop”), then following course of arch in postmedial line’s anterior part and meeting with dorsum at about two thirds of dorsum length; apical streak more or less pronounced, narrow; ends of veins on dorsum in some specimens with minute dark brown dots, but often missing; fringe light cream-white. Hindwing in males cream-white to brownish, postmedial line brown, more or less clear, terminal band brown, often only at apex; hindwing in females evenly dark brown, some specimens with a slightly darker terminal band. Underside of forewing uniformly brown with a slightly darker distal discoidal stigma and, in some specimens, with a slightly darker postmedial line; underside of hindwing greyish white to grey, with costal area somewhat darker, a diffuse brownish grey postmedial line might be visible in males, in females a more or less pronounced terminal band is present.

*Abdomen*. Dorsal side of abdomen covered with cream-white glossy scales, ventral side with dark brown scales, interspersed by cream-white scales; male 8^th^ segment with long beige posterior scales. Tympanum with broad short lobulus; 2^nd^ sternite with broad U-shaped sclerotisation, more so in females, inner part less sclerotised; 3^rd^ sternite anterior edge with broad sclerotised lobe on each side of the centre, tapering anterolaterad into thin apex; 4^th^ sternite anterior edge with oval hole in sternite sclerotisation on each side of the centre; centre of anterior edge of sterites V-VII with broad, short rectangular protrusion; male 8th sternite with U-shaped sclerotisation along borders, posterior ends broadened; male 8th tergite with broad central longitudinal sclerotisation, somewhat broadening anteriorly, leading laterally into pointed triangular process.

*Male genitalia*. (Figs 23–28) Uncus broadly attached to tegumen, the attachment site laterally constricted; apical part of uncus constricted into slim, strap-like neck leading into flattened oval uncus head, dorsally covered densely with stiff, deeply bifid setae. Tegumen broad, rectangular. Broad, weakly sclerotised gnathos band with a central conical dorsad protrusion. Transtilla arms forming equilateral triangles, dorsal surface sparsely set with thin long simple setae. Vinculum broad, well sclerotised, sides elongate drop-shaped to oval, ventral part mediodorsally forming broad bell-shaped protrusion towards juxta, ventrally forming U-shaped saccus with prominent ventromedial keel. Juxta nearly rhomboidal to almost circular, apex sharply bifid with medial incision about one quarter as long as juxta. Valva long, slender, slightly tapering towards apex; costa slightly concave, broader at base, surface sparsely set with thin, long, simple setae; sacculus broad, roughly oval, dorsodistal edge close to fibula base, ventrodistal part concavely curving towards ventral valva edge; ventral valva edge straight apart from a slightly concave recess in the area of the fibula tip; valva apex rounded towards distal end of costa; slender, evenly broad, strongly sclerotised fibula directed towards sacculus apex, apical half narrowed to pointed, ventrad curved claw, ventral side of claw flat; fibula emerging from oval sclerotised lobe near base of costa, sparsely set with thin, long simple setae. Phallus tubular, slightly curved, evenly sclerotised; posterior phallus apodeme bent sinistrad, dorsally and ventrally with elongate unsclerotised strip, right posterior phallus apodeme forming sclerotised spatulate lobe with medially protruding ridge containing three (rarely two) teeth of variable shape apically.

*Female genitalia*. (Fig. 45) Corpus bursae membranous, oval; elongate lens-shaped longitudinal signum extending through corpus bursae, 3.4- to 4.4-times as long as broad (n = 8). Ductus bursae membranous apart from short sclerotised section in posteriormost part and oval, longitudinally folded auxiliary signum in anterior section where it transitions into corpus bursae; length of auxiliary signum 29–60% of main signum length (n=8); ductus bursae anteriorly broad, tapering posteriad to half its diameter; posterior section and colliculum less strongly sclerotised, with thickened mesocuticle, colliculum forming short ‘S’; ductus seminalis membranous, emerging at colliculum. Antrum broad, tubular to conical, strongly sclerotised, without thickened mesocuticle; length of sclerotisation equal to antrum diameter (longer in dissected female from Korab), with variably pronounced unsclerotised longitudinal strip in antrum’s dorsal sclerotisation. Postvaginal area membranous, sparsely covered with evenly spread tiny spicules. Segment 8 a broad, strongly sclerotised, ventrally open band, with anterior side broadly recessed where apophyses anteriores attach; apophyses anteriores slender, with broadened triangular to rhomboidal muscle attachment area at ca. 1/3 of their length. Apophyses posteriores slender, ca. 2/3 as long as apophyses anteriores. Papillae anales simple, with long setae on outer margin and shorter ones elsewhere.

##### Immature stages.

Not studied and, to our knowledge, not described in the literature.

##### Distribution.

**Figures 4–5.**
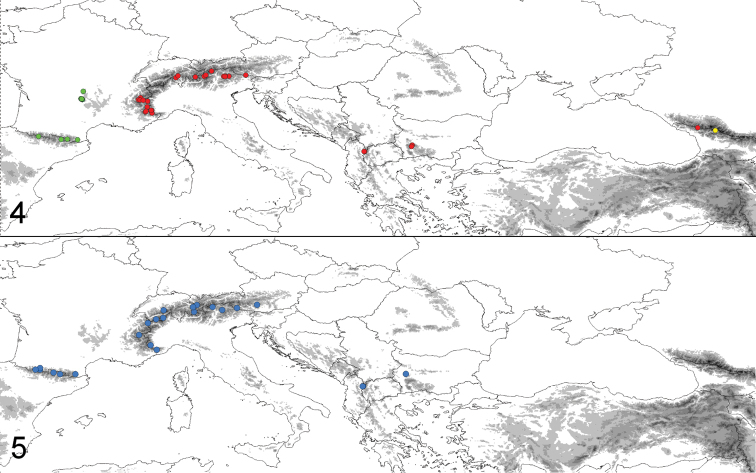
Distribution of investigated specimens of the *Udea austriacalis* species complex (**4**) and *U. rhododendronalis* (**5**) in Europe **4** *U. austriacalis* (red), *U. donzelalis* (green), *U. cretacea* (yellow) **5** *U. rhododendronalis* (blue); altitudes ≥ 1,000 m are marked in increasingly darker grey shades every 500 m.

Alps, Southern Balkan and Caucasus, where it might occur sympatrically with *U. cretacea* (Fig. 4).

##### Food plants.

Lhomme (1935) reports *U. austriacalis* from *Plantago major* L. (Plantaginaceae), and the synonymous *“Pyrausta” sororialis* Heyden, 1860 as polyphagous.

##### DNA data.

See Table 2. In BOLD, *U. austriacalis* is represented by the BINs AAD2363 and AAD2364. The seven DNA barcoded specimens forming the clade from the Central and East Alps and Macedonia differ between 0% and 0.95% in p-distance, whereas the nine specimens from the Maritime Alps are identical in their DNA barcodes. The two clades are closest to each other in COI p-distances, with a minimum of 2.38%; furthermore, the Maritime Alps clade has *C. cretacea* as closest nearest COI neighbour (see Tab. 3). The next-closest neighbours are *U. rhododendronalis*, *U. uliginosalis* and *U. ruckdescheli* with a minimum interspecific COI p-distance of 4.29%. *Udea donzelalis* (see below) differs by 5.24–5.71% COI p-distance from *U. austriacalis*.

##### Remarks.

The type material was not stated in the original description of Herrich-Schäffer (1847–1855 [“1849”]), however, on pl. 20 fig. 142 a male imago is illustrated. Type material is also not stated in Herrich-Schäffer (1843–1856 [“1856”]).

#### Udea donzelalis

Taxon classificationAnimaliaLepidopteraCrambidae

(Guenée, 1854) stat. rev.

Figs 1 , 2–3 , 4 , 11–14 , 29–33 , 46

Scopula donzelalis Guenée, 1854: 392, pl. 6 fig. 12.

##### Type locality.

France, Auvergne-Rhône-Alpes region, Département Puy-de-Dôme, Arrondissement Clermont-Ferrand, Mont-Dore.

##### Material examined.

**Type specimens. Lectotype** ♀ “Puy de Dôme | Mont Dore | Guenée”, [orange label] “Cotype”, [circular label with yellow margin] “Co- | type”, “Paravicini Coll. | B. M. 1937-383.”, NHMUK loan label NHMUK010589059, [salmon-pink label] “DNA voucher | Lepidoptera | ZMBN 2017 | [transverse] no. 473”, Mally prep. no. 1123 ♀ (NHMUK); **3 Paralectotypes**: 1♂ with labels as Lectotype, NHMUK loan label NHMUK010589061, [salmon-pink label] “DNA voucher | Lepidoptera | ZMBN 2017 | [transverse] no. 474”, Mally prep. no. 1124 ♂ (NHMUK); 1♂ with labels as Lectotype plus [brown label] “Donzelalis | Gn. Mont Dore”, NHMUK loan label NHMUK010589062, [salmon-pink label] “DNA voucher | Lepidoptera | ZMBN 2017 | [transverse] no. 475”, Mally prep. no. 1125 ♂ (NHMUK); 1♀ [abdomen missing] “Puy de Dôme | Mont Dore | Guenée”, [orange label] “Cotype”, [circular label with yellow margin] “Co- | type” (NHMUK).

##### Additional material

**(aus1 i Fig. 1): FRANCE.** 1♂ “Plomb du Cantal | 13-7-2007 | D. Tourlan”, [yellow label] “DNA voucher | Lepidoptera | ZMBN 2015 | [transverse] no. 089”, Mally prep. no. 1022 (coll. D. Tourlan); 1♂ [handwritten] “Le Lioran | Puy Griou | CANTAL | 10.07.1988 | D. TOURLAN” (coll. D. Tourlan); 1♂ same data but with date “25.6.1989” (coll. D. Tourlan); 1♂ [handwritten] “Le Lioran | Bataillouze | CANTAL | 10.07.1991 | D. TOURLAN” (coll. D. Tourlan); 1♂ “S.-Frankr.,Pyr.or. | Mt. Canigou | 12–16.VI.’24.Zerny” (NHMW); 1♂ “Le Lioran | CANTAL | [handwritten] 25.6.2011 | D. Tourlan”, [yellow label] “DNA voucher | Lepidoptera | ZMBN 2015 | [transverse] no. 091”, Mally prep. no. 1024 (coll. D. Tourlan); 2♀ same collection data but with date “28.7.1997” and “25.7.2001”, Mally prep. no. 1103 & 1104 (coll. D. Tourlan); 1♀ “Pas de Peyrol | CANTAL | [handwritten] 31.7.2010 | D. Tourlan”, [yellow label] “DNA voucher | Lepidoptera | ZMBN 2015 | [transverse] no. 090”, Mally prep. no. 1023 (coll. D. Tourlan); 1♀ [handwritten] “Gavarnie | H.Pyr. France | 15. VII. 1958 | [printed] S.N.A.JACOBS.”, “Brit. Mus. | 197[handwritten]2-305””, Mally prep. no. 1047 (NHMUK); 1♀ [handwritten] “Col de Puy Morens | Pyr.Or. France | 26. VII. 1958 | [printed] S.N.A.JACOBS.”, “Brit. Mus. | 197[handwritten]2-305”, Mally prep. no. 1048 (NHMUK); 1♂ 1♀ “Pyrénées Orientales | Mt. Canigou | Bellier”, Mally prep. no. 1102 (♀) (NHMUK); **ANDORRA.** 3♂ “ANDORRA | Port de Cabús, 2290 m | 1°25'13´E [sic], 42°32'45"N | 16.7.2012 | leg. Huemer | TLMF 2012-011”, [yellow label] “DNA voucher | Lepidoptera | ZMBN 2014 | [transverse] no. 084” and “085”, Mally prep. no. 866 and 867 (TLMF); 5♂ “ANDORRA | Port de Cabús, 2290 m | 1°25'13"E, 42°32'45"N | 16.7.2012 | leg. Huemer | TLMF 2012-011”; **SPAIN.** 1♂ “Spain, Huesca | Balneario de Panticosa | 42.75, -0.217, 1650 m | 14.07.2012, leg. P. Huemer”, “BC TLMF Lep 20010” (TLMF).

##### Diagnosis.

Labial palps in males approx. 20% longer than in *U. austriacalis* and *U. cretacea*; outer side of labial palps’ 2^nd^ and 3^rd^ palpomeres barely darker than rest of labial palps. Maculation of forewing usually more pronounced than in *U. austriacalis*, the apical brown streak often broader; hindwing in both sexes with greyish cream-white inner area contrasted by a prominent dark brown postmedial line and dark brown terminal band, especially in specimens where the postmedial line and the outer band are fused into a broad band; in contrast, the hindwings’ upper side is evenly dark brown in females of *U. austriacalis*. In the male genitalia, the fibula of *U. donzelalis* is generally broader and elongate triangular, tapering from the broad base towards the apex; in *U. austriacalis* the fibula is narrower and evenly broad from the base to the subapex. In the female genitalia, the signum of *U. donzelalis* is 4.1–5.1 times as long as broad (n = 6), whereas in *U. austriacalis* the signum is 3.4–4.4 times as long as broad (n = 8) (Tab. 1). *Udea donzelalis* can furthermore be distinguished by the DNA Barcode from all other sequenced *Udea* species; the nearest neighbour is *U. cretacea* with 4.29% minimum p-distance.

##### Redescription.

*Head*. As for *U. austriacalis*, apart from: frons and vertex covered with cream-white to beige scales; labial palps covered with light brown scales, outer sides of labial palps’ second and third segment sometimes slightly darker with dirty light brown scales; maxillary palps approximately one third as long as labial palps, with beige to light brown apical scale tuft; antennal length approx. 60 % of forewing length in males, approx. 70 % in females.

*Thorax*. As for *U. austriacalis*, apart from: legs cream-white except for dark brown inner side of fore- and midlegs; hindleg proximal outer spur ca. 2/3 length of inner spur, distal spurs almost equal in length, outer slightly shorter.

*Wings*. Forewing length 12–13 mm in males, 8–10 mm in females. Males and females with one frenulum bristle. Female forewing with more acute apex due to the straight costa (distally curved in males). Forewing ground colour glossy cream-white, with maculation more or less prominent: a light brown streak parallel to the basal two thirds of the costa, distal discoidal stigma a diffuse brownish area, postmedial line brownish, arching around distal discoidal stigma, then turning basad until below distal discoidal stigma, sharply arching back outwards (the “*Udea* loop” typical for most species in the genus), then following the course of the arch in the postmedial line’s anterior part and meeting with the dorsum at about two thirds of the dorsum length; apical streak more or less pronounced, usually relatively broad; ends of veins on dorsum with minute dark dots; fringe light cream-white. Hindwing in both sexes cream-white to grey-brown, postmedial line and terminal band dark brown, can be fused into one broad band. Underside of forewing uniformly brown, sometimes with grey-grown strip along cell; underside of hindwing greyish white with a brown tinge, postmedial line brownish grey, rather diffuse; colour of external area as internal area, or brownish grey as postmedial line, with which it can form a broad band.

*Abdomen*. As for *U. austriacalis*.

*Male genitalia*. (Figs 29–33) As for *U. austriacalis*, apart from: juxta nearly rhombical to broad drop-shaped, apex sharply bifid with medial incision about one fifth of juxta length; ventral valva edge straight to slightly convex; elongate triangular, apically tapering, strongly sclerotised fibula directed towards distal sacculus apex, apical half narrowed to pointed, ventrad curved claw, ventral side of claw flat; right posterior phallus apodeme forming sclerotised spatulate lobe with medially protruding ridge containing three teeth of varying shape at posterior end.

*Female genitalia*. (Fig. 46) As for *U. austriacalis*, apart from: signum 4.1- to 5.1-times as long as broad (n = 6); length of auxillary signum 42–62 % of main signum length (n=6); length of antrum sclerotisation 1–1.5 times the antrum diameter.

##### Immature stages.

Unknown.

##### Distribution.

Massif Central (France), Pyrenees (France, Andorra, Spain) (Fig. 4).

##### Food plants.

Unknown.

##### DNA data.

See Table 2. On BOLD, *U. donzelalis* is represented by BIN ADB6837. The five DNA barcoded specimens are identical in their DNA barcodes. The nearest neighbour is *U. cretacea* with 4.29 % minimum p-distance in the COI Barcode (see Tab. 3). The next-closest neighbour is *U. rhododendronalis* with a minimum interspecific COI p-distance of 4.76 %.

##### Remarks.

Lederer (1863) synonymised *donzelalis* with *U. austriacalis*, and following authors (La Harpe 1864, Marion 1973, Leraut 1996) came to the same conclusion. However, Leraut (1996) mentioned that the specimens from the Pyrenees, conspecific with the revised *U. donzelalis*, differ from other specimens of *U. austriacalis* in their clearer maculation on the forewings in both sexes. Based on the investigation of the four syntypes of *U. donzelalis*, a lectotype and three paralectotypes are designated (see material examined).

#### Udea altaica

Taxon classificationAnimaliaLepidopteraCrambidae

(Zerny, 1914) stat. n.

Figs 6 , 15–18 , 36–37 , 47

Pyrausta austriacalis v. altaica Zerny, 1914: 334–335.

##### Type locality.

Mongolia, central Altai mountains.

##### Material examined.

**Type specimens. Lectotype** ♀ “Altai centr. | mont.”, “Stgr. | [handwritten] 1914”, “667.”, [handwritten] “P. austriacalis | v. altaica | Zerny ♀ [in red] Type”, Mally prep. no. 1084 (NMW); **Paralectotype** ♂ (abdomen lost) “Altai centr. | mont.”, “Stgr. | [handwritten] 1914”, “666.”, [handwritten] “*P. austriacalis* | v. altaica | Zerny ♂ [in red] Type” (NMW). – **Additional material. MONGOLIA.** 3♂ 2♀ “Altai”, one of the ♂ also with [handwritten] “Alticolalis | BH i L”, Mally prep. no. 1099 (♀), 1100 (♀), 1117–1119 (♂) (ZMHB); 1♂ 1♀ [handwritten] “Pyrausta | Alticolalis | Altai BH”, Mally prep. no. 1090 (♂) & 1098 (♀) (ZMHB).

##### Diagnosis.

Proximal outer spur of hindleg minute (as in *U. alpinalis*, *U. juldusalis* and *U. plumbalis*), whereas in *U. austriacalis*, *U. cretacea*, *U. donzelalis* and *U. uliginosalis* it is ca. half to two thirds the length of the proximal inner spur. The wing maculation of *U. altaica* can be confused with that of *U. austriacalis*, *U. cretacea*, *U. donzelalis*, *U. juldusalis*, *U. plumbalis*, *U. uliginosalis* and untypically maculated specimens of *U. alpinalis* (see Fig. 4 in Panigaj and Kulfan 2012), but it can be distinguished from all those species by the more or less distinct proximal brown section of the postmedial line on the ventral side of the forewing in both sexes (Figs 16, 18); in males, the proximal subterminal area of the ventral forewing side is as light brown as the central area (Fig. 16), whereas in the other species it is darker than the central area; on the hindwing ventral side, the subterminal area is only faintly darker than the central wing area in both sexes (Figs 16, 18), whereas the other species have a darker subterminal area, at least in the apex. In male genitalia only distinguishable from *U. cretacea* and *U. uliginosalis* by the small dentate ridge-like process on the posterior phallus apodeme, whereas in *U. cretacea* the sclerotisation at posterior phallus apodeme is a slim, elongate, apically dentate process emerging from the posteriormost end (Fig. 35), in *U. uliginosalis* a large hooked spine. In the female genitalia, the main signum is 3.1- to 3.4-times as long as broad, whereas in *U. austriacalis* the main signum is 3.4- to 4.4-times longer and in *U. donzelalis* 4.1- to 5.1-times longer than its maximum width (Tab. 1). The antrum is conical, widening posteriorly and is about twice as long as broad (Fig. 47), whereas in *U. austriacalis* and *U. donzelalis* the sclerotised antrum is predominantly tubular and 1- to 1.5-times as long as broad (Figs 45–46).

##### Redescription.

*Head*. As for *U. austriacalis*, part from: frons and vertex with cream-white scales; distal half of labial palps brown on the outside, basal half and inner sides cream white; maxillary palps cream-white with some brown scales mixed in; antennal length approx. 60 % of forewing length in males, approx. 70 % in females.

*Thorax*. As for *U. austriacalis*, apart from: legs cream-white on inner and outer sides; proximal pair of metatarsal spurs with outer spur minute and inner spur long, distal pair ca. half the length of the proximal inner spur, distal inner spur a bit longer than distal outer spur.

*Wings*. Forewing length 14 mm in males, 11 mm in females. Males and females with one frenulum bristle. Female forewing with more acute apex due to the straight costa (distally curved in males), and with outer wing margin (termen) of hindwing cut straight. Forewing upper side cream white with brown scales interspersed, giving it a dirty appearance; brownish subcostal line along the basal two third of the forewing; cell margin facing the forewing centre demarcated by a thin brown line, less prominent in females; outer medial area with a transverse cream white band lacking the interspersed brown scales; outer margin of cream white band delimited by diffuse grey postmedial line which leaves the costa in a right angle, bends inward at vein M1 and parallels the termen until the line reaches the dorsum; postmedial area homogenous greyish white, apex with more or less darker streak; termen with a slim brown margin and long, cream-white fringes. Hindwing upper side in males pale yellowish brown with diffuse light brown apex, in females light brown with a faint, slightly brighter medial band. Forewing underside in females vivid brown, with a darker, diffuse outer cellular spot and a darker postmedial area, demarcated by the postmedial line, maculation paler in males; slim whitish subcostal line along the basal two third of the forewing; fringes cream-white. Hindwing underside cream white with the subcostal and terminal areas tinted slightly brownish, the postmedial line more or less prominent.

*Abdomen*. Pale grey dorsally, slightly darker grey ventrally; distal segment margins greyish white, scales on terminal segment pale yellowish. Tympanum without broad short lobulus.

*Male genitalia*. (Figs 36–37) As for *U. austriacalis*, apart from: juxta nearly rhombical to broad drop-shaped, with small indention on each side dorsal of its greatest width, apex sharply bifid with narrow V-shaped medial incision ca. 1/4 of juxta length; ventral valva edge convex, with a slight bulge in the area to which the fibula is pointing; valva apex evenly rounded. An elongate triangular, apically tapering, strongly sclerotised fibula directed towards the distal sacculus, apical half narrowed to pointed, ventrad curved claw, ventral side of claw with flat ‘blade’; fibula emerging from an oval sclerotised lobe near base of costa which is very sparsely studded with thin long simple setae; posterior phallus apodeme dorsally and ventrally with elongate unsclerotised strip, right posterior phallus apodeme forming a sclerotised spatulate lobe with a medially protruding ridge containing at the posterior end two equal-sized teeth and a third tiny posterior-most tooth.

*Female genitalia*. (Fig. 47) As for *U. austriacalis*, apart from: signum 3.1- to 3.4-times as long as broad (n = 4); auxillary signum 52–67 % length of main signum (n=4); antrum conical, widening posteriorly, sclerotised section about twice as long as its diameter, with or without a narrowly V-shaped unsclerotised longitudinal indentation in the antrum’s dorsodistal sclerotisation; apophyses posteriores slender, approx. 60–80 % the length of the apophyses anteriores.

##### Immature stages.

Unknown.

##### Distribution.

**Figure 6.**
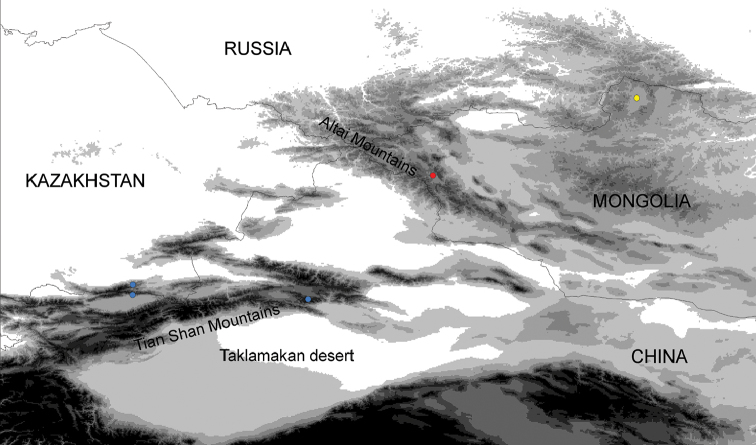
Distribution of investigated specimens of *Udea juldusalis* (blue), *U. altaica* (red) and *U. plumbalis* (yellow) in Central Asia; altitudes ³ 1,000 m are marked in increasingly darker grey shades every 500 m, altitudes ³ 4,000 m are in black. Note that the eastern locality of *U. juldusalis* and the localities of *U. altaica* and *U. plumbalis* are only approximations of the type localities.

The species is known from the central Altai mountains in NW Mongolia, the Küngöy Ala-Too (Kungey Alatau) Range in Kazakhstan and Kyrgyzstan, and the Yulduz mountains in NW China (see Fig. 6); the Küngöy Ala-Too and Yulduz mountain ranges are part of the Tian Shan mountains.

**Figures 7–14.**
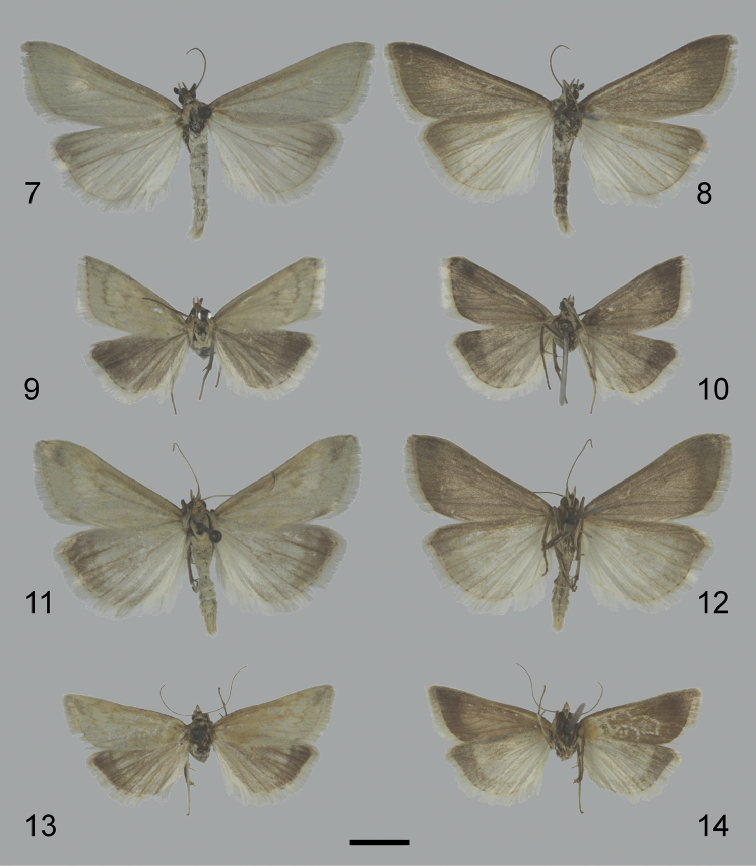
Adult specimens of *Udea* species. **7–10** *U. austriacalis* **7–8** male, dorsal (**7**) and ventral (**8**) **9–10** female, dorsal (**9**) and ventral (**10**), abdomen removed **11–14** *U. donzelalis* **11–12** male, dorsal (**11**) and ventral (**12**) **13–14** female, dorsal (**13**) and ventral (**14**), abdomen removed. Scale bars: 500 µm.

**Figures 15–22.**
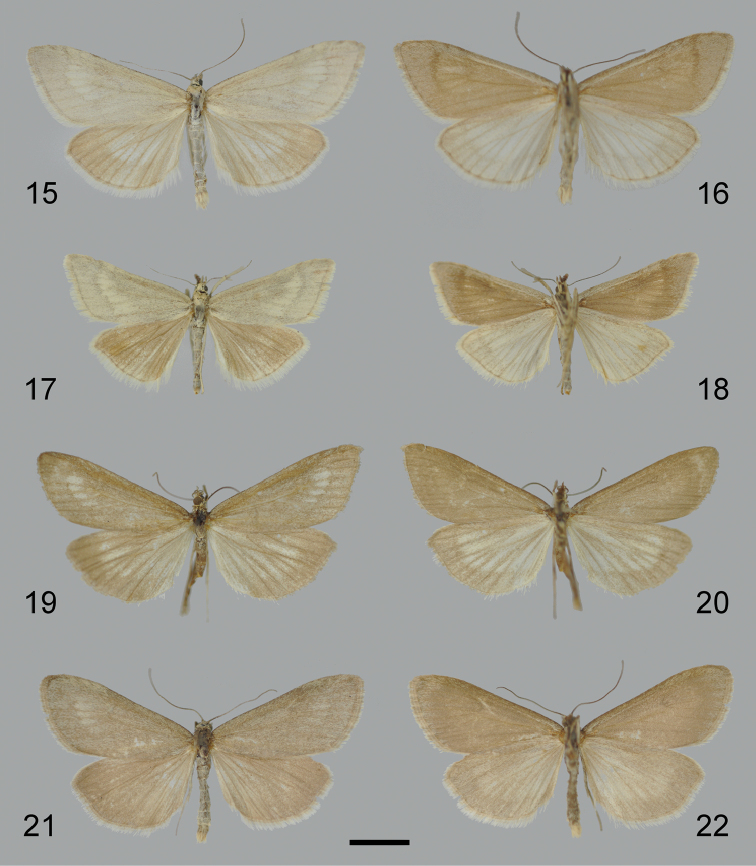
Adult specimens of *Udea* species. **15–18** *U. altaica* **15–16** male, dorsal (**15**) and ventral (**16**) **17–18** Lectotype (NHMW) female, dorsal (**17**) and ventral (**18**) **19–20** *U. juldusalis* Lectotype (NHMW) male, dorsal (**19**) and ventral (**20**) **21–22** *U. plumbalis* Holotype (NHMW) male, dorsal (**21**) and ventral (**22**). Scale bar: 500 µm.

**Figures 23–35.**
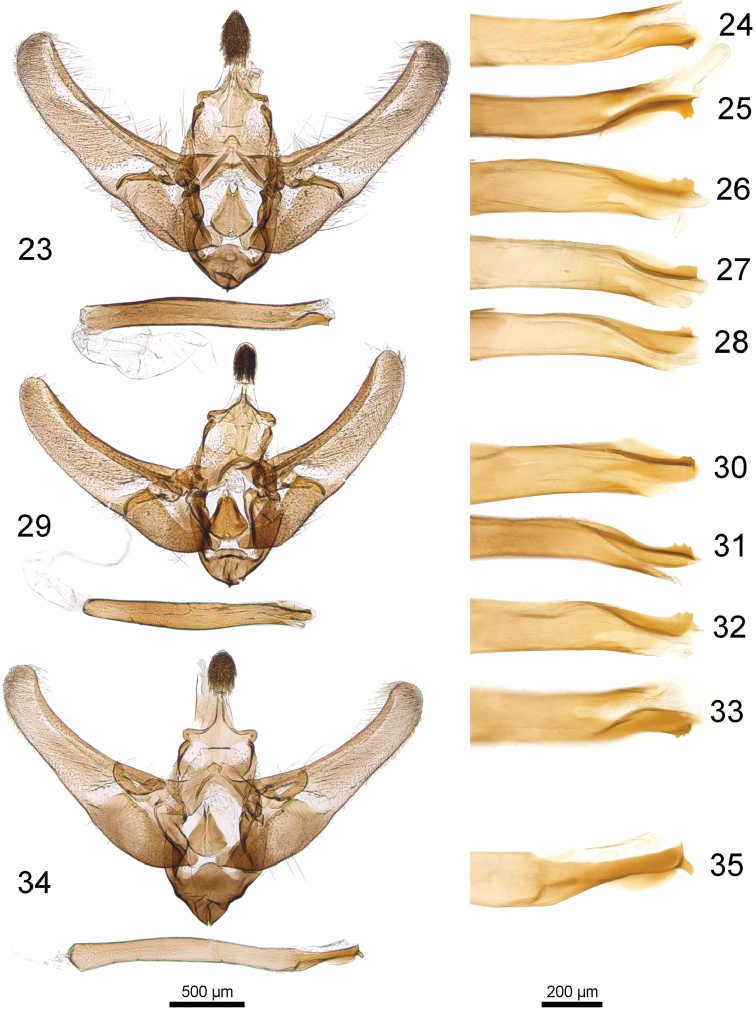
Male genitalia of the *Udea austriacalis* species complex. **23–28** *U. austriacalis* **23** male genitalia (Mally prep. 1092) **24–28** posterior phallus apodeme **24** Mally prep. 1042 **25** Mally prep. 1043 **26** Mally prep. 1044 **27** Mally prep. 1045 **28** Mally prep. 1046 **29–33** *U. donzelalis* **29** male genitalia (Mally prep. 1024) **30–33** posterior phallus apodeme **30** Mally prep. 1024 **31** Mally prep. 866 **32** Mally prep. 867 **33** Mally prep. 1022 **34–35** *U. cretacea* (Mally prep. 523) **34** male genitalia **35** posterior phallus apodeme; 500 µm scale bar refers to male genitalia, 200 µm scale bar to posterior phallus apodemes.

**Figures 36–44.**
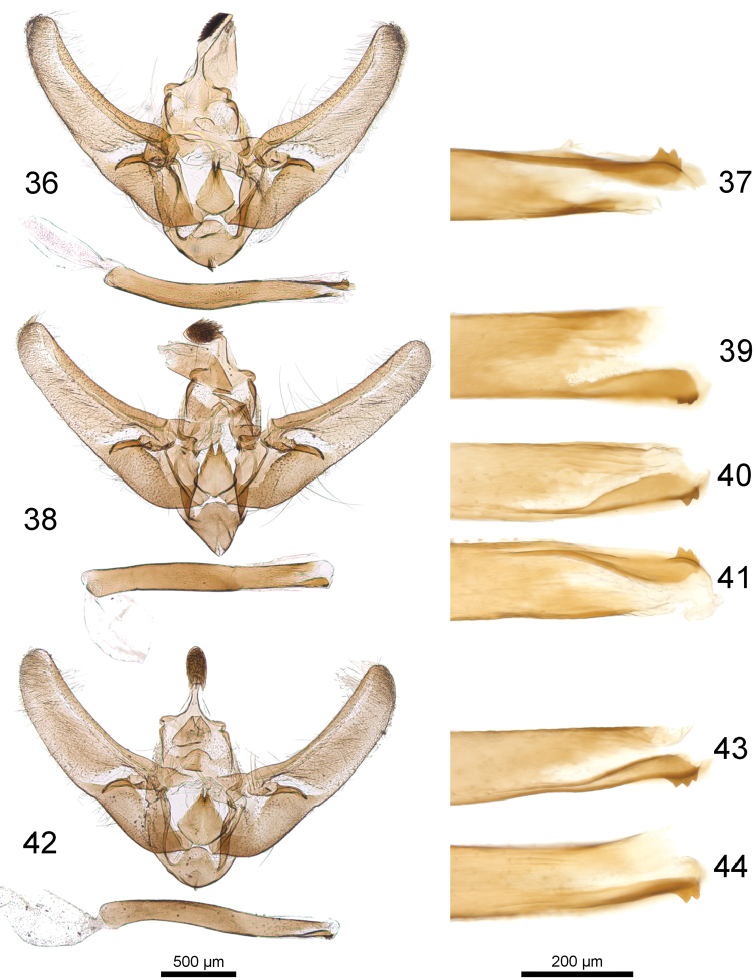
Male genitalia of *Udea* species. **36–37** *U. altaica* (Mally prep. 1090) **36** male genitalia **37** posterior phallus apodeme **38–41** *U. juldusalis* **38** male genitalia, Paralectotype (Mally prep. 1081) **39–41** posterior phallus apodeme **39** Paralectotype (Mally prep. 1081) **40** Lectotype (Mally prep. 1082) **41** (Mally prep. 1089) **42–44** *U. plumbalis* **42** male genitalia, Holotype (Mally prep. 1083) **43–44** posterior phallus apodeme **43** Holotype (Mally prep. 1083) **44** (Mally prep. 1094); 500 µm scale bar refers to male genitalia, 200 µm scale bar to posterior phallus apodemes.

**Figures 45–47.**
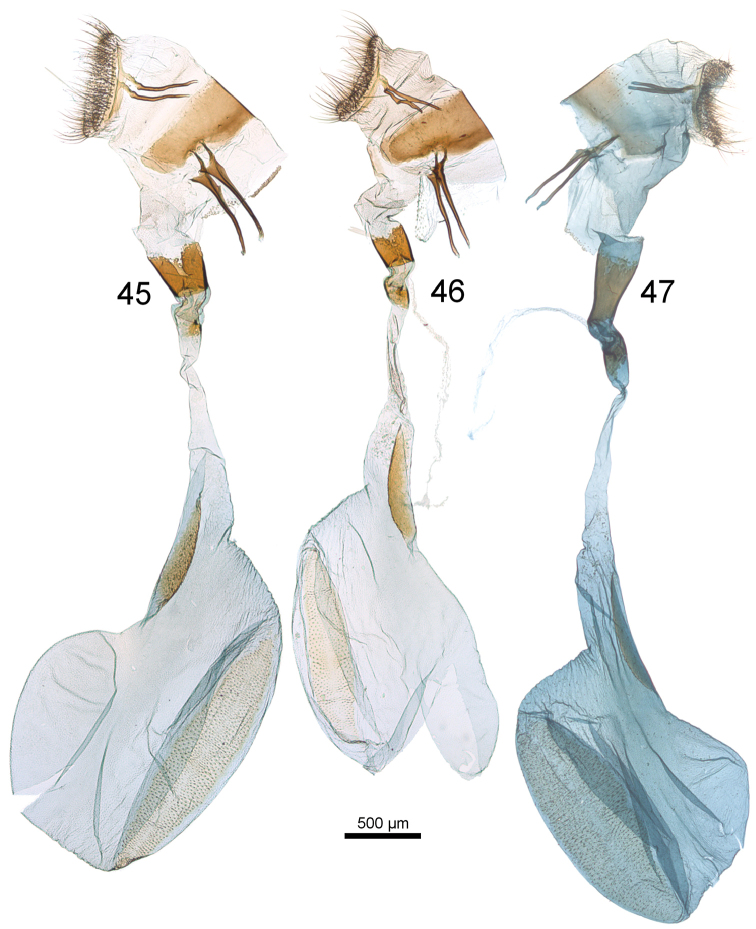
Female genitalia of *Udea* species. **45** *U. austriacalis* (Mally prep. 1047) **46** *U. donzelalis* (Mally prep. 1023) **47** *U. altaica* (Mally prep. 1084).

##### Food plants.

Unknown.

##### DNA data.

Unavailable.

#### Udea juldusalis

Taxon classificationAnimaliaLepidopteraCrambidae

(Zerny, 1914) stat. n.

Figs 1 , 6 , 19–20 , 38–41

Pyrausta austriacalis v. juldusalis Zerny, 1914: 335.

##### Type locality.

China, Xinjiang, Tian Shan, Yulduz mountains.

##### Material examined.

**Type specimens. Lectotype** ♂ “Asia centr. | Thian-Schan | Juldus Geb. | Coll.Wagner”, [handwritten] “P. austriacalis | v. juldusalis | Zerny ♂ [in red] Type”, Mally prep. no. 1082 (NMW); **Paralectotype** ♂ [handwritten] “Thian-Schan | Juldus Geb | Coll. Wagner”, Mally prep. no. 1081 (NMW).

##### Additional material.

**CHINA.** 1♂ [handwritten] “Pyrausta | Plumbealis | v. Juldusalis | Juldus BH.”, Mally prep. no. 1089 (ZMHB); **KYRGYZSTAN.** 1♂ “Kyrgyzstan, Ysyk-Kol, Chong Oruktu, 42.796 77.86, 1900 m, 22-Jun-1998, L. Kuehne”, [light green label] “DNA Barcode | BC MTD 00522”, Mally prep. no. 1126 (MTD); **KAZAKHSTAN.** 1♂ “Kazakhstan, Almaty Oblysy, Zailijskij Alatau, Turgen valley, 43.217 77.867, 2660 m, 19-Jun-2000, M. Nuss” [light green label] “DNA Barcode | BC MTD 00523”, Nuss prep. no. 1127 (MTD).

##### Diagnosis.

*Udea juldusalis* has a wing maculation similar to that of *U. altaica*, *U. plumbalis*, *U. uliginosalis* and untypically maculated specimens of *U. alpinalis* (see Fig. 4 in Panigaj and Kulfan 2012). It can be distinguished from *U. uliginosalis* by the minute proximal outer spur of the hindleg, which is well developed in *U. uliginosalis* and about half to two thirds the length of its proximal inner spur; furthermore, *U. uliginosalis* has a large hooked spine on the posterior phallus apodeme, whereas the posterior phallus apodeme carries a small dentate ridge-like process in *U. juldusalis*. *Udea altaica* specimens have lighter forewings dorsally and ventrally, and the proximal section of the postmedial line is a diffuse, though well visible brown arch in both sexes. *Udea plumbalis* has darker, broader and more rounded fore- and hindwings (at least in the male). *Udea alpinalis* is distinguished by the dark subterminal band on the hindwings’ dorsal and ventral side that contrasts with the white inner hindwing area. The COI sequences (DNA Barcode) of *U. juldusalis* are unique and not shared with any other DNA-barcoded organism, and the nearest neighbour is *U. uliginosalis* with a minimum of 2.86 % p-distance.

##### Redescription.

*Head*. Frons and vertex covered with cream-white scales; frons evenly convex, covered with beige to light brown scales; labial palps projecting forward, third segment pointed, palps covered with beige scales, outer sides of labial palps’ second and third segment brown; maxillary palps brown on outside, cream-white on the inside apart from subapical area with brown scaling; compound eyes hemispherical; proboscis well developed, its base covered in brown and greyish scales; antennae filiform, dorsal side covered in beige scales, anterior side in males densely covered with cilia shorter than the basal antennal radius, shorter still in females, antennal length approx. 50% of forewing length in males, females unknown; ocellus posterior to antenna base.

*Thorax*. As for *U. austriacalis*, apart from: greyish brown ground colour; hindlegs and outer side of fore- and midlegs cream-white, inner side of fore- and midleg brown; proximal pair of metatarsal spurs with outer spur minute and inner spur long, distal pair about half the length of the proximal inner spur, distal inner spur a bit longer than distal outer spur.

*Wings*. Forewing length 13–14 mm in males, females unknown. Males with single frenulum bristle. Forewing dorsal side pale brownish to brownish yellow white, somewhat darker between cell and costa; veins delimiting cell pale brown; outer medial area behind cell cream white and clear, intersected by the brownish coloured veins R5, M1–3 and Cu1, outer margin of cream white area sharply defined by postmedial line; postmedial line slightly darker than brown ground colour, indistinct at anterior and posterior wing margins, smoothly curving around whitish area of medial wing; outer wing margin with two thin brownish lines separated by a thin yellowish cream-white line; distal part of fringe pale whitish. Hindwing dorsal side with dirty white to brownish ground colour, intersected by the brown-tinted veins; a broad brown subterminal band along the outer margin, blurrily demarcated from the somewhat lighter inner area; hindwing outer margin with a thin yellowish line between subterminal band and brownish basal half of fringe; distal half of fringe whitish. Forewing ventral side homogenous brown, subcostal area and veins delimiting the cell somewhat darker; more or less prominent white line along costal side of cell; outer wing margin and fringe as on dorsal side. Hindwing ventral side with dirty white basal and central area, intersected by the brown-tinted veins; brown subterminal band more prominent and clearly marked-off from the inner area; outer wing margin and fringe as on dorsal side.

*Abdomen*. As for *U. austriacalis*, apart from: abdomen pale grey dorsally, dark grey ventrally; distal segment margins on dorsal side cream white, scales on terminal segment pale yellow.

*Male genitalia*. (Figs 38–41) As for *U. austriacalis*, apart from: vinculum ventrally forming a roundly V-shaped saccus with a prominent ventromedial keel; juxta nearly rhombical to broad drop-shaped, apex sharply two-pointed with a narrow V-shaped medial incision ca. 1/4 of the juxta length; valva long, relatively broad, slightly tapering towards apex; costa concave, evenly tapering towards apex, surface sparsely studded with thin long simple chaetae; sacculus broad, roughly rectangular, dorsodistal edge close to fibula base, ventrodistal part concavely curving towards ventral valva edge; ventral valva edge straight from mid-sacculus to valva subapex, with a very slight bulge in the area where the fibula is pointing to; valva apex evenly rounded; elongate, apically tapering, strongly sclerotised fibula directed towards the distal sacculus, fibula base somewhat constricted, apical half narrowed to a pointed, ventrad curved claw; right posterior phallus apodeme forming a sclerotised spatulate lobe with a central raised ridge bearing two small, laterally protruding teeth at its posterior end, sometimes with one or two additional, much smaller teeth posterior to the larger ones.

##### Female genitalia.

Unknown.

##### Immature stages.

Unknown.

##### Distribution.

The species is known from the Küngöy Ala-Too (Kungey Alatau) Range in Kazakhstan and Kyrgyzstan, and from the Yulduz mountains in NW China; both mountain ranges are part of the Tian Shan mountain system (Fig. 6).

##### Food plants.

Unknown.

##### DNA data.

See Table 2. On BOLD, *U. juldusalis* is represented by the BIN AAO4297. The two available DNA Barcodes are identical with each other. The nearest COI Barcode neighbour is *U. uliginosalis* with 2.86–4.29 % p-distance (see Tab. 3); the next-closest neighbour is *U. alpinalis* with 3.33–4.29 % p-distance.

#### Udea plumbalis

Taxon classificationAnimaliaLepidopteraCrambidae

(Zerny, 1914) stat. n.

Figs 6 , 21–22 , 42–44

Pyrausta austriacalis v. plumbalis Zerny, 1914: 335.

##### Type locality.

Mongolia, Khövsgöl Province, eastern Sayan mountains, Darhad basin, Arsain Gol river.

##### Material examined.

**Type specimen. Holotype** ♂ “Arasagun-gol | Sajan”, “Stgr. | [handwritten] 1914”, [handwritten] “Pyrausta | plumbealis B.H.iL.”, [handwritten] “P. austriacalis | v. plumbalis | Zerny ♂ [in red] Type”, Mally prep. no. 1083 (NMW). – **Additional material. MONGOLIA.** 1♂ “Arasagun-gol | Sajan”, [handwritten] “Pyrausta | Plumbealis | [crossed out “Juldus”] BH.”, Mally prep. no. 1094 (ZMHB).

##### Diagnosis.

*Udea plumbalis* can be confused with *U. juldusalis*, *U. uliginosalis*, *U. uralica* and untypically maculated specimens of *U. alpinalis* (see Fig. 4 in Panigaj and Kulfan 2012). It differs from *U. uliginosalis* in the minute proximal outer spur of the hindleg, which is well developed in *U. uliginosalis*; furthermore, *U. uliginosalis* has a large hooked spine on the posterior phallus apodeme, while *U. plumbalis* has a small dentate ridge-like process. In *U. alpinalis*, the forewing apex is more acute and the hindwings have a white inner area (with a more or less broad brown area along the dorsum, sometimes occupying the majority of the inner wing area) contrasted with a clear dark-brown terminal band along the termen, whereas in *U. plumbalis* the hingwings’ dorsal side is evenly brown (Fig. 19), and on the ventral side the inner wing area is only faintly lighter than the subterminal band (Fig. 20). *Udea juldusalis* has lighter, narrower, and more acute fore- and hindwings in the male, and the hindwings’ inner area is lighter. *Udea plumbalis* is distinguished from *U. uralica* by the rounded apex and dark fringe in the forewing of males, and by the absence of a prominent bulge in the centre of the ventral valva edge (compare Slamka 2013, pl. 27 fig. 129); it is not clear whether this bulge on the ventral valva edge or its absence is a reliable character to distinguish the two species; the length of the hindlegs’ proximal outer spur in comparison to the proximal inner spur is not known for *U. uralica*.

##### Redescription.

*Head*. As for *U. austriacalis*, apart from: frons and vertex with greyish brown scales; proboscis base covered in brown and greyish scales; labial palps directed forward, dirty- to cream white, distal half of the outside greyish brown; maxillary palps brown on outside, cream white on the inside; dorsal side of antennae with line of metallic brown scaling; antennal length approx. 60 % of forewing length in males, females unknown.

*Thorax*. As for *U. austriacalis*, apart from: greyish brown thorax; front legs and inner side of mid- and hindlegs cream-white, side of mid- and hindlegs pale brownish with many cream-white scales intermixed; hindleg proximal outer spur minute, inner spur the longest one on the hindleg, distal outer spur ca. 80% the length of the inner spur.

*Wings*. Forewing length 12–13 mm in males, females unknown. Males with one frenulum bristle. Forewing dorsal side dirty pale brown, distal of the postmedial line brown-grey to dirty greyish. Maculation absent apart from a more or less prominent oval whitish area basal of the postmedial line on the M veins. Area between costa and cell and veins encircling the cell somewhat darker brown. Slim band along outer wing margin consisting of three thin brown lines alternated with two thin pale yellowish brown lines; distal fringe pale white. Hindwing pale brown, apical area somewhat darker; outer wing margin with thin bands as in forewing, outermost band somewhat fainter. Ventral side of forewing uniformly brown, costa blended with darker brown and greyish scales and with a thin whitish line running in parallel on the length of the cell; distal end of cell with a faint darker brown arc; subterminal area slightly darker; dirty greyish brown area on wing apex, running along outer wing margin, narrowing towards posterior end of termen; outer wing margin and fringe as on dorsal side. Ventral side of hindwing dirty brownish grey with a more or less prominent broad pale brown subterminal band; outer wing margin and fringe as on dorsal side.

*Abdomen*. As for *U. austriacalis*, apart from: abdomen brownish grey, distal segment margins on dorsal side and scales on terminal segment pale yellow.

*Male genitalia*. (Figs 42–44) As for *U. austriacalis*, apart from: juxta rhombical to broad drop-shaped, apex sharply bifid with a narrow V-shaped medial incision 20–25% of the juxta length; Valva long, relatively broad to slim, slightly tapering towards apex; costa concave, central costa somewhat narrowed, costa surface sparsely studded with thin long simple setae; sacculus broad, roughly rectangular, dorsodistal edge close to fibula base, ventrodistal part concavely curving towards ventral valva edge; ventral valva edge straight to convex, with or without a slight bulge in the area where the fibula is pointing to; fibula elongate, apically tapering, strongly sclerotised, directed towards the distal sacculus, dorsal fibula edge inflated to a narrow tube, apical half narrowed to a pointed, ventrad curved claw, ventral side of claw with flat surface; right posterior phallus apodeme forming a sclerotised spatulate lobe with a central raised ridge bearing two to three small, laterally protruding teeth at its posterior end.

##### Female genitalia.

Unknown.

##### Immature stages.

Unknown.

##### Distribution.

So far only known from the eastern Sayan mountains in the Khövsgöl Province in N Mongolia (Fig. 6).

##### Food plants.

Unknown.

##### DNA data.

No data available.

#### Udea rhododendronalis

Taxon classificationAnimaliaLepidopteraCrambidae

(Duponchel, 1834)

Figs 1 , 2–3 , 5

Botys rhododendronalis Duponchel, 1834: 363–364, pl. 235 fig. 5.
= Udea rhododendronalis luquetalis P. Leraut, 1996: 216–217, syn. n.
= Udea rhododendronalis ventosalis P. Leraut, 1996: 215–216, syn. n.

##### Material examined.

**Alpine DNA Barcode clade (BOLD BIN AAH7703; rho2 in Fig. 1): FRANCE.** 1♂ “France, Alpes | Maritimes, 2000m | 6 km NW Tende | Mont Chajol | 5.vii.2008 | O. Karsholt”; “ZMUC | 00400001”; [salmon-pink label] “DNA voucher | Lepidoptera | ZMBN 2016 | [transverse] no. 413”, Mally prep. no. 1035 (ZMUC); 1♂ “Htes. Alpes | Lauteret | Fletcher Coll. | [handwritten] 31.VII. 1932” (BMNH); **SWITZERLAND.** 1♂ “Heuthal | Graubuenden | [handwritten] 4.VIII.1902”, “Coll. E. Möbius | Ankauf 1946”, Mally prep. no. 47 (MTD); 1♂ [handwritten] “Pontresina | Switz. | 12.VII.1965 | [printed] S.N.A.JACOBS.”, “Brit. Mus. | 197[handrwritten]2 305” (BMNH); 1♂ “Valais | Arolla | 6500 ft. | [handwritten] 5.Aug 1925 | Fletcher coll.”, [handwritten] “Pyrausta | rhododendronalis, Dup | (V.1594)” (BMNH); 1♂ 1♀ “Switzerland. | [underlined]Scheidegg- | Wengen. | 8. vii. 1948 | S.N.A.Jacobs”; “Brit. Mus. | 197[handwritten]2-305”, Mally prep. no. 1030 ♀ & 1031 ♂ (BMNH); 1♂ 1♀ [handwritten] “SaasFee | Switz. | 26.VI.1967 | [printed] S.N.A.JACOBS”, “Brit. Mus. | 197[handwritten]2 305” (BMNH); 1♂ 1♀ “Graubünden | Davos, Dorfthäli | Pfr. Hauri”, “Paravicini Coll. | B.M. 1937-383.” (BMNH); **AUSTRIA.** 1♂ [red-bordered label, handwritten] “Tirol | Ötztal | 22.8.26 | Starke | Bautzen | [transverse] 21ov”, “Coll. STARKE / Bautzen | Ankauf 1953 | Übernahme 1969”, Mally prep. no. 5 (MTD); 1♂ “Austria, Osttirol | Obertilliach | Golzentipp, 2070–2317m | 01.07.2007 | leg. A. Stübner”, [orange label] “DNA voucher | Lepidoptera | M. Nuss. 2007 | [transverse] no. 242”, Mally prep. no. 56 (MTD); 2♂ “Austria merid., Steiermark | Turracher Höhe NW | 1750–1850 m | 13°52'05"E, 46°55'41"N | 4.7.2009 | leg. Huemer”, [pale green label] “ BC TLMF Lep 00899” and “00900”, one ♂ with [salmon-pink label] “DNA voucher | Lepidoptera | ZMBN 2016 | [transverse] no. 414”, Mally prep. no. 1036 (TLMF); 1♂ “Austria, Vorarlberg | Partenen, 2,5 km W | Silvrettastausee, 1980 m | 10°03'43"E, 46°55'21"N | 3.7.2010, leg. Huemer | TLMF 2010-020”, [turquoise label] “BC TLMF Lep 09148”, [salmon-pink label] “DNA voucher | Lepidoptera | ZMBN 2016 | [transverse] no. 415”, Mally prep. no. 1037 (TLMF); 1♀ [red-bordered label, handwritten] “Tirol | Ötztal | 22.8.26 | Starke | Bautzen | [transverse] 22ov”, “Coll. STARKE / Bautzen | Ankauf 1953 | Übernahme 1969”, Mally prep. no. 6 (MTD); **ITALY.** 1♂ [handwritten] “Südtirol, Grödner Tal | Plan dla Gran Costa | nördl.St.Ulrich 2150 m | 9.7.1990 leg Sutter”; [handwritten] “[transverse] 253 | ♂ Udea | det.R.Sutter | Dup | rhododendronalis”; [light green label] “DNA Barcode | BC MTD 00792”, [orange label] “DNA voucher | Lepidoptera | ZMBN 2014 | [transverse] no. 116”, Mally prep. no. 1025 (SMNK); 1♂ “Italien, Prov. Cuneo | Alpi Cozie, Demonte NW | Gias Valcavera | 7°8,2'E, 44°22,6'N | 2050 m, 23.7.2009 | leg. Huemer | TLMF 2009-138”; [turquoise label] “BC TLMF Lep 00972”, [salmon-pink label] “DNA voucher | Lepidoptera | ZMBN 2016 | [transverse] no. 417”, Mally prep. no. 1039 (TLMF); 1♀ “ITALIA, Südtirol, | Pfelders, Pfelderer Alm, | 11°03'28’’ E, 46°46'50’’ N | 1850 m, 25.-26.6.2010 | leg. Huemer”; [turquoise label] “BC TLMF Lep 09218”, [salmon-pink label] “DNA voucher | Lepidoptera | ZMBN 2016 | [transverse] no. 416”, Mally prep. no. 1038 (TLMF); 1♂ [handwritten] “Courmayeur | Aosta Italy | 13.VI.1964. | [printed] S.N.A.JACOBS.”, “Brit. Mus. | 197[handwritten]2 305” (BMNH).

##### Pyrenean and Cantabrian DNA Barcode clade

**(BOLD BIN ABZ6001; rho1 in Fig. 1): SPAIN.** 1♂ “ESPANA, Prov. Cantabria | PN Picos de Europa | Fuente De, El Cable Bergst. | 4°48,53´W, 43°09,55´N | 1870 m, 11.7.2012 | leg. Huemer | TLMF 2012-011”, [light red label] “DNA voucher | Lepidoptera | R. Mally 2012 | [transverse] no. 1390”, Mally prep. no. 560 (TLMF); 1♂ same data, without orange DNA voucher label, Mally prep. no. 569 (TLMF); 1♂ same data, but [yellow label] “DNA voucher | Lepidoptera | ZMBN 2014 | [transverse] no. 075”, Mally prep. no. 870 (TLMF); 1♂ same data, but [yellow label] “DNA voucher | Lepidoptera | ZMBN 2014 | [transverse] no. 076”, Mally prep. no. 871 (TLMF); 2♂ “Spain, prov. Lerida | 42°39'13’’ N, 00°60 E | East of Port de la | Bonaigua, 1925 m | 31.vii.2007 | leg. B.Skule & P.Skou”, [salmon-pink label] “DNA voucher | Lepidoptera | ZMBN 2016 | [transverse] no. 418”, Mally prep. no. 1040 (ZMUC); **ANDORRA.** 1♂ “ANDORRA | Port de Cabús, 2290 m | 1°25'13´[sic!]E, 42°32'45"N | 16.7.2012 | leg. Huemer | TLMF 2012-011”; [light red label] “DNA voucher | Lepidoptera | R. Mally 2012 | [transverse] no. 1391”, Mally prep. no. 561 (TLMF); 1♂ same data, but [yellow label] “DNA voucher | Lepidoptera | ZMBN 2014 | [transverse] no. 074”, Mally prep. no. 869 (TLMF); **FRANCE.** 1♂ [handwritten] “Franz. Zentralpyrenäen | Res.Nat. Neouvielle | Lac d’Aumar | 2200m; 15.VIII.1991 | [printed] leg.M. Sommerer”, [pale turquoise label] BC TLMF Lep 05660”, Mally prep. no. 1032 (TLMF); 1♂ [handwritten] “NEL Jacques | Lac de Gaube | 65.Cauterets. PN | 28.VII.2002”, “gen ♂ | 14454” (TLMF); 1♂ 1♀ [handwritten] “20.7.94” (♂) and [handwritten] “1.7.47” (♀), “Pyrénées Orientales | Vernet-les-Bains | R. Oberthür”, “Paravicini Coll. | B.M. 1937-383.”, Mally prep. no. 1107 (♂), 1108 (♀) (BMNH); 1♀ [handwritten] “NEL Jacques | Pic Midi Bigorre | 2350m.65 | 07.VIII.2002”, [yellow label] “DNA voucher | Lepidoptera | ZMBN 2015 | [transverse] no. 088”, Mally prep. no. 873 (TLMF); 1♀ [handwritten] “NEL Jacques | Pic Midi Bigorre | 2350m.65 | 07.VIII.2002”, [handwritten] “gen ♀ | 14437” (TLMF).

##### Balkan DNA Barcode clade

**(rho3 in Fig. 1): MACEDONIA.** 1♂ “Macedonia, NP Mavrovo | Korab, summit ridge | ca. 2700–2750 m | 20°32'48"E, 41°47'20"N | 28.7.–1.8.2011 | leg. Huemer & Tarmann”, [pale green label] “BC TLMF Lep 05086”, [yellow label] “DNA voucher | Lepidoptera | ZMBN 2014 | [transverse] no. 073”, Mally prep. no. 868 (TLMF); 1♀ “Macedonia, NP Mavrovo | Korab, eastern ridge | ca. 2325–2400 m | 20°34'46"E, 41°47'08"N | 28.7.–1.8.2011 | leg. Huemer & Tarmann”; [pale green label] “BC TLMF Lep 05082”, Mally prep. no. 848 (TLMF); **BULGARIA.** 4♂ 3♀ “Bulg. Vitoscha | 2290 m,23.7.83 | leg.J Ganev”; “Brit. Mus. | 198[handwritten]5∙189” or “Brit. Mus. | 198[handwritten]5∙282”, Mally prep. no. 1026 ♀, 1027 ♂, 1110 ♂ (BMNH).

##### DNA data.

See Table 2. On BOLD, *U. rhododendronalis* is represented by the BINs AAH7703 and ABZ6001.

##### Remarks.

We conclude from the data that none of the genitalia characters stated by Leraut (1996) for his subspecies are diagnostic: The shape of the valva apex and the size and extent of the protruding tooth ridge on the posterior phallus apodeme is variable in *U. rhododendronalis* (and in other species of the *U. alpinalis* species group); the number of cornuti ranges from four to six and is not fixed for any of the proposed subspecies. In the female genitalia, the width of the ductus bursae is variable and can be significantly influenced by the uptake of spermatophore(s) during copulation. One of the dissected females contained two spermatophores in its genital tract, indicating the possibility of multiple mating. Due to the absence of distinct morphological characters among the three clades, the two subspecies *U. r. luquetalis* and *U. r. ventosalis*, both described by Leraut (1996), are synonymised with *U. rhododendronalis*.

The *U. alpinalis* species group now consists of the following taxa (in alphabetical order):

*Udea alpinalis* (Denis & Schiffermüller, 1775)

= *Phalaena grisealis* Fabricius, 1794

= Pyrausta alpinalis ab. prolongata Weber, 1945

= *Pyrausta alpinalis valerialis* Galvagni, 1933

= *valerianalis* Speidel, 1996 (misspell.)

*Udea altaica* (Zerny, 1914), **stat. n.**

*Udea austriacalis* (Herrich-Schäffer, 1851)

= *Botys nitidalis* Heinemann, 1865

= *Botys sororialis* Heyden, 1860

*Udea bourgogealis* Leraut, 1996

*Udea carniolica* Huemer & Tarmann, 1989

*Udea cretacea* (Filipjev, 1925)

*Udea donzelalis* (Guenée, 1854), **stat. rev.**

*Udea juldusalis* (Zerny, 1914), **stat. n.**

*Udea murinalis* (Fischer von Röslerstamm, 1842)

*Udea nebulalis* (Hübner, 1796)

= *Botys pratalis* Zeller, 1841

= *Pyralis squalidalis* Hübner, 1809

*Udea plumbalis* (Zerny, 1914), **stat. n.**

*Udea rhododendronalis* (Duponchel, 1834)

= *Udea rhododendronalis luquetalis* P. Leraut, 1996, **syn. n.**

= *Udea rhododendronalis ventosalis* P. Leraut, 1996, **syn. n.**

*Udea uliginosalis* (Stephens, 1834)

= *Pyrausta monticolalis* La Harpe, 1855

= *uliginosalis* (Stephens, 1829), **nom. nud.**

*Udea uralica* Slamka, 2013

## Discussion

Deep intraspecific splits are observed in the results of a phylogenetic analysis of COI sequence data of the *U. alpinalis* species group. However, further investigation of these clades based on nuclear genetic and morphological data does not indicate species-specific differences, with the exception of the *U. austriacalis* clade from the French Massif Central and the Pyrenees. We conclude that the latter clade represents a species distinct from *U. austriacalis*, as it differs in morphology and in all three investigated genetic markers from *U. austriacalis*. This distinct species is identified as *U. donzelalis*, after comparison with the respective type material, and consequently revoked from synonymy with *U. austriacalis*.

In the COI phylogram (Fig. 1), the *U. rhododendronalis* clade rho1 corresponds with the geographic distribution of Leraut’s (1996) subspecies *U. rhododendronalis luquetalis*, but no morphological character could be found to separate it from the nominate *U. rhododendronalis* from the Alps. Similarly, the Balkan specimens of *U. rhododendronalis*, forming clade rho3 in the COI phylogram, are morphologically not distinct from the specimens of the Alps and Pyrenees, and thus not supporting Slamka’s (2013) assumption of as potential Balkan subspecies. Specimens from the Maritime Alps, from where Leraut’s (1996) subspecies *U. r. ventosalis* is described, do not form a distinct COI clade, like the *U. r. luquetalis* specimens, but group with the *U. rhododendronalis* specimens from the Central Alps instead. Furthermore, the COI groups are not reflected in the phylogenetic results of the two nuclear markers (Figs 2–3). In conclusion, no conclusive evidence for either additional species or subspecies in *U. rhododendronalis* is found.

Morphological investigation of the type material of Zerny’s (1914) three *U. austriacalis* subspecies reveals that they are good species, and they are consequently raised to species level. For *U. juldusalis*, this decision is further supported by COI Barcode sequences which are not shared with other *Udea* species.

Similar cases of deep intraspecific, although not necessarily allopatric, divergences in COI data of Lepidoptera have been observed in other groups, e.g. by Charlat et al. (2009), Dasmahapatra et al. (2010), Muñoz et al. (2011), Mutanen et al. (2012), Nieukerken et al. (2012) and Ritter et al. (2013). In *U. rhododendronalis* and *U. austriacalis*, geographically well-separated COI groups are found. The observed mitochondrial genetic divergences could therefore be explained with allopatry, and the absence of (congruent) clades in the nuclear data could be due to slower substitution rates and incomplete lineage sorting. However, the specimens of *U. donzelalis* form distinct, congruent clades among all three investigated genetic markers. No such congruent pattern is found for the COI subclades of *U. austriacalis* and *U. rhododendronalis*, respectively, and the species status is therefore rejected for the named subclades of *U. rhododendronalis* based on the available data.

The uniformity of male genitalia in the *U. alpinalis* species group makes species discrimination based on this character complex difficult to impossible, as is also the case for example in the *U. fimbriatralis* complex (Mally et al. 2016). The female genitalia, however, are suitable for species distinction: the sclerotised parts of antrum, colliculum and signa as well as the ratio of length to breadth of the main signum are useful for species identification. Munroe (1966) points out the usefulness of the hindleg’s proximal outer spur for distinguishing species of the *U. itysalis* group, a character that is also useful for distinguishing species of the *U. alpinalis* group.

### Introgression between *U. alpinalis* and *U. uliginosalis*

Some specimens morphologically identified as *U. uliginosalis* have been found grouping with specimens of *U. alpinalis* in the COI Barcode ML analysis (Fig. 1). All those ‘mismatched’ specimens are males, and their genitalia present the diagnostic characters of *U. uliginosalis*. Analyses of two nuclear markers result in different topologies: The ML analysis of wingless data (Fig. 2) does not provide a differentiation between *U. alpinalis* and *U. uliginosalis*. In the ML analysis of EF1-alpha data (Fig. 3), all ‘mismatched’ specimens are monophyletic with all other specimens of *U. uliginosalis* and are sister to *U. alpinalis*. The contradicting placements of those ‘mismatched’ *U. uliginosalis* specimens in the mitochondrial and nuclear ML analyses leads us to the assumption that introgression must have occurred between *U. uliginosalis* and *U. alpinalis*. This could be explained by the following scenario: A female of *U. alpinalis* successfully mates with a male of the sympatrically occurring *U. uliginosalis*. The F1 generation has the mitochondrial genotype of *U. alpinalis*, and the nuclear genotype is heterozygotic. In concordance with Haldane’s rule (Haldane 1922), the sex that is heterogametic for sex factors – in Lepidoptera this is the female – is rare or sterile or absent. The specimens of *U. uliginosalis* collected at the localities of assumed hybridisation are exclusively male, without a single female. (However, females are less frequently collected than males - they may be poor flyers due to their shorter wings.) Despite this, assuming that a few fertile hybrid females exist in the F1 generation and these mate with males of *U. uliginosalis*, the F2 (backcrossing) generation retains the mitochondrial genotype of *U. alpinalis*, since the mitochondrial genome is maternally inherited; the nuclear genotype of the offspring consists to three quarters of that of *U. uliginosalis*. Additional backcrossings further increase the proportion of the *U. uliginosalis* nuclear genome, while the mitochondrial genotype remains that of *U. alpinalis*.

The deep intraspecific splits led us to test for *Wolbachia* infection which might play a role in the *U. alpinalis* group. The intracellular bacterium *Wolbachia* is known to have an impact on the reproduction of a wide range of arthropods, including Lepidoptera (e.g. Werren et al. 2008). The observed deep intraspecific COI Barcode splits in species of the *U. alpinalis* group could be explained by *Wolbachia*-mediated cytoplasmic incompatibility between different populations, or by geographic isolation, or a mix of both processes. In the present screening, no evidence of a *Wolbachia* infection was found in specimens of the *U. alpinalis* species group. However, several instances of false negative results exist that could obscure the presence of *Wolbachia* in these species: The mean *Wolbachia* infection prevalence is 21 % among Crambidae (Ahmed et al. 2015), so that *Wolbachia* can remain undetected if the sampling of a population is not comprehensive enough. The amplification with wsp primers resulted in sequences from *U. fulvalis* and *U. olivalis* that could not be matched with *Wolbachia* or any other sequences in GenBank or BIGSdb, indicating suboptimal primer sequences which can lead to failure of PCR amplification or amplification of fragments other than the target sequence. Although no evidence for the presence of *Wolbachia* in the *U. alpinalis* group was found in the present screening, the possibility of *Wolbachia* infection and its biological implications for the hosts should be kept in mind for future studies.

The analyses of the two nuclear markers (Figs 2–3) show partially contradictory results: in the wingless gene tree (Fig. 3), a relatively long terminal branch leads to the *U. donzelalis* clade, whereas in the EF1a gene tree (Fig. 2), the branch of the same clade is much shorter. Further, in the EF1a phylogram *U. uliginosalis* and *U. alpinalis* form separate clades, whereas in the wingless phylogram the specimens of those two species share a common clade. This indicates that more nuclear markers are required to reliably reconstruct this contradictory and insufficiently supported part of phylogenetic inference.

These results shed further light on the *U. alpinalis* species group, but more material is needed from the mountain systems of Central and West Asia to study the morphology and genetics of the species found there, to bring them in phylogenetic context with European species of the *U. alpinalis* group and to investigate the biogeography of the species group. Additional morphological investigations are encouraged regarding the status of the genetic clades. In a similar case, new techniques, like the eversion of the phallus vesica, allowed the morphological differentiation of hitherto exclusively genetic clades (Zlatkov and Huemer 2017).

Other *Udea* species groups require revisionary work, like the Palaearctic *U. numeralis* group that needs the most systematic attention in this region. Mally et al. (2016) described a new species from Crete and placed it in the *U. numeralis* group, based on a phylogenetic analysis, but several problematic taxa remain to be revised in this group, e.g. the *U. fimbriatralis* complex and the *U. numeralis* complex as well as *U. praepetalis* (Lederer, 1869) and *U. bipunctalis* (Herrich-Schäffer, 1848). Future systematic studies on this group should include material from the Middle East and the East Palaearctic, especially the taxa described by Amsel (1961, 1970). In North America, the majority of the 25 *Udea* species belong to the *U. itysalis* group (Munroe 1966) that requires careful revision with the help of molecular data. A large COI Barcode dataset for Nearctic *Udea* material has been accumulated, but analysis of the data is pending (pers. comm. Jean-François Landry). Apart from these studies, *Udea* is poorly investigated in systematic terms. With this integrative revision, the number of *Udea* species is raised from 217 to 221 species, with 40 of them occurring in Europe.

## Supplementary Material

XML Treatment for Udea austriacalis

XML Treatment for Udea donzelalis

XML Treatment for Udea altaica

XML Treatment for Udea juldusalis

XML Treatment for Udea plumbalis

XML Treatment for Udea rhododendronalis
